# Opposing Activities of LIT-1/NLK and DAF-6/Patched-Related Direct Sensory Compartment Morphogenesis in *C. elegans*

**DOI:** 10.1371/journal.pbio.1001121

**Published:** 2011-08-09

**Authors:** Grigorios Oikonomou, Elliot A. Perens, Yun Lu, Shigeki Watanabe, Erik M. Jorgensen, Shai Shaham

**Affiliations:** 1 Laboratory of Developmental Genetics, The Rockefeller University, New York, New York, United States of America; 2 Howard Hughes Medical Institute, Department of Biology, University of Utah, Salt Lake City, Utah, United States of America; UC Berkeley, United States of America

## Abstract

Glial cells surround neuronal endings to create enclosed compartments required for neuronal function. This architecture is seen at excitatory synapses and at sensory neuron receptive endings. Despite the prevalence and importance of these compartments, how they form is not known. We used the main sensory organ of *C. elegans*, the amphid, to investigate this issue. *daf-6*/Patched-related is a glia-expressed gene previously implicated in amphid sensory compartment morphogenesis. By comparing time series of electron-microscopy (EM) reconstructions of wild-type and *daf-6* mutant embryos, we show that *daf-6* acts to restrict compartment size. From a genetic screen, we found that mutations in the gene *lit-1*/Nemo-like kinase (NLK) suppress *daf-6*. EM and genetic studies demonstrate that *lit-1* acts within glia, in counterbalance to *daf-6*, to promote sensory compartment expansion. Although LIT-1 has been shown to regulate Wnt signaling, our genetic studies demonstrate a novel, Wnt-independent role for LIT-1 in sensory compartment size control. The LIT-1 activator MOM-4/TAK1 is also important for compartment morphogenesis and both proteins line the glial sensory compartment. LIT-1 compartment localization is important for its function and requires neuronal signals. Furthermore, the conserved LIT-1 C-terminus is necessary and sufficient for this localization. Two-hybrid and co-immunoprecipitation studies demonstrate that the LIT-1 C-terminus binds both actin and the Wiskott-Aldrich syndrome protein (WASP), an actin regulator. We use fluorescence light microscopy and fluorescence EM methodology to show that actin is highly enriched around the amphid sensory compartment. Finally, our genetic studies demonstrate that WASP is important for compartment expansion and functions in the same pathway as LIT-1. The studies presented here uncover a novel, Wnt-independent role for the conserved Nemo-like kinase LIT-1 in controlling cell morphogenesis in conjunction with the actin cytoskeleton. Our results suggest that the opposing *daf-6* and *lit-1* glial pathways act together to control sensory compartment size.

## Author Summary

The nervous system of most animals consists of two related cell types, neurons and glia. A striking property of glia is their ability to ensheath neuronal cells, which can help increase the efficiency of synaptic communication between neurons. Sensory neuron receptive endings in the periphery, as well as excitatory synapses in the central nervous system, often lie within specialized compartments formed by glial processes. Despite the prevalence of these compartments, and their importance for neuronal function and signal transmission, little is known about how they form. We have used the amphid, the main sensory organ of the worm *Caenorhabditis elegans*, to investigate glial sensory compartment morphogenesis. We demonstrate that the glia-expressed gene *daf-6*/Patched-related acts to restrict the size of the sensory compartment, while the Nemo-like kinase *lit-1* acts within glia in the opposite direction, to promote sensory compartment expansion. We show that LIT-1 localizes to the sensory compartment through a highly conserved domain. This domain can interact both with actin, which outlines the compartment, and with the regulator of actin polymerization WASP, which acts in the same pathway as *lit-1*. We postulate that Nemo-like kinases could have broader roles as regulators of cellular morphogenesis, in addition to their traditional role in regulating the Wnt signaling pathway.

## Introduction

Sensory organs are the gates through which information flows into the nervous system. In many sensory organs, specialized glial cells form a chemically isolated compartment around neuronal receptive endings [1],[2]. For example, in the skin, the mechanosensory Pacinian corpuscles consist of an unmyelinated nerve ending that is surrounded by lamellae formed by a modified Schwann glial cell [3]. In the olfactory epithelium, sensory neurons are ensheathed by glia-like sustentacular cells [4],[5]. In the inner ear, hair cells are surrounded by Deiter's cells, which express the glial marker glial fibrillary acidic protein (GFAP) [6]; and in the vertebrate eye, retinal pigmented epithelial cells contact photoreceptor cell cilia [7]. At least in some cases, the integrity of the glial compartment is essential for proper sensory neuron function [8]. Glial compartments also enclose excitatory neuronal synapses in the cerebellum and hippocampus [9],[10], and are thought to be important for synaptic function through limiting neurotransmitter diffusion, and regulating levels of synaptic effectors. Despite the prevalence of such glial compartments, little is known about their development.

**Figure 1:**
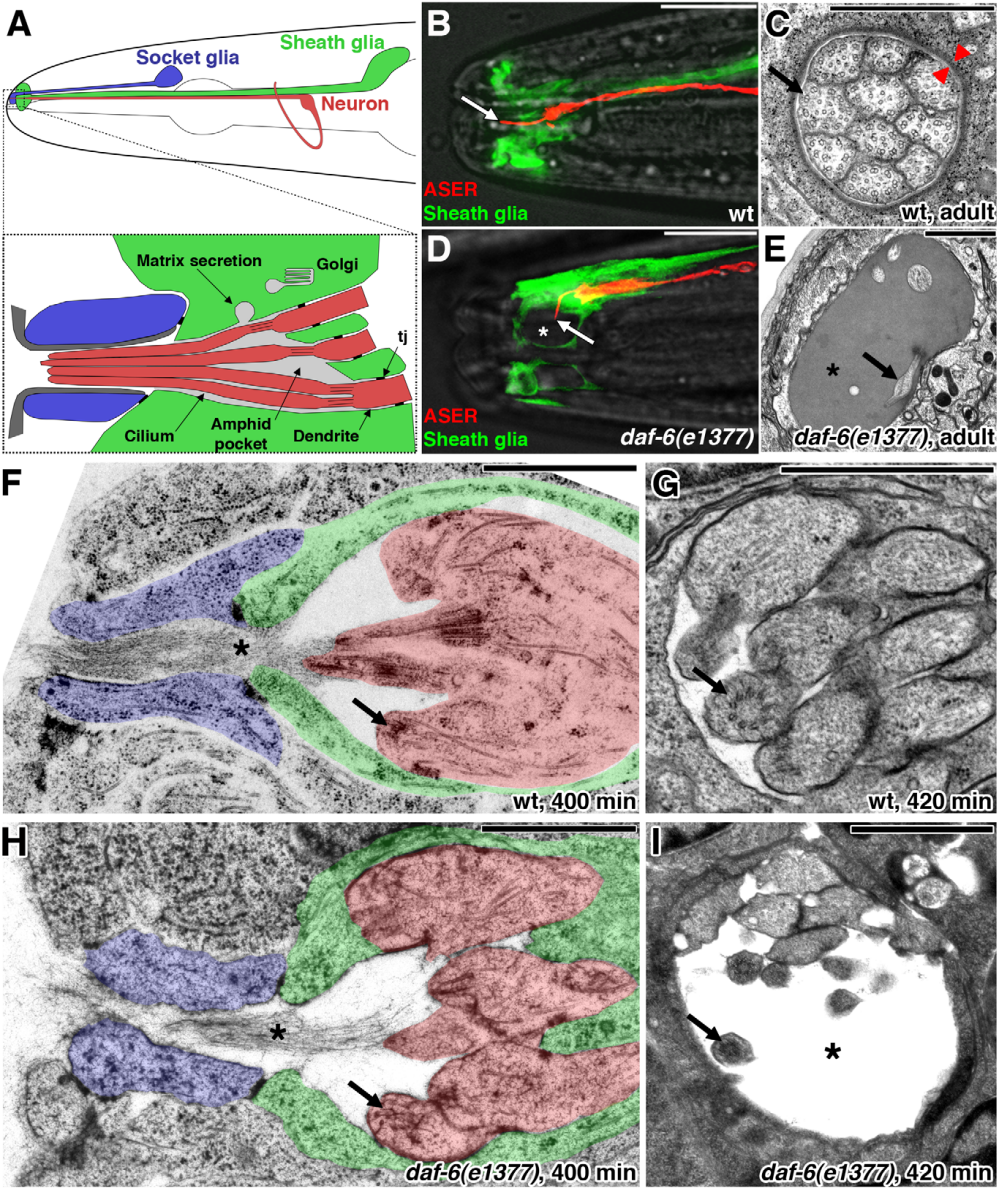
*daf-6* restricts amphid sensory compartment size. In longitudinal sections and diagrams (A, B, D, F, and H) anterior is left. White scale bars, 10 µm. Black scale bars, 1 µm. (A) Schematic of the *C. elegans* amphid. Top: Each amphid consists of 12 neurons (only one is depicted here) and two glial cells, the sheath and the socket. Bottom: Detail of the anterior tip of the amphid. Matrix is secreted by the Golgi apparatus. tj, tight junction. Adapted from [13]. (B, D) The ASER neuron and the amphid sheath glia visualized, respectively, with mCherry (red; driven by the *gcy-5* promoter) and GFP (green; driven by the *T02B11.3* amphid sheath promoter [32] in a wild-type (B) or *daf-6*(*e1377*) (D) animal (transgenes *nsEx2766* and *nsEx2752*, respectively)). The ASER neuron extends a single cilium through the length of the amphid channel in the wild type (arrow). In the mutant, the cilium is bent and not exposed to the environment, and the amphid pocket is bloated (asterisk). (C, E) Electron micrograph of a cross-section through the anterior portion of the amphid sheath glia channel in an adult wild-type animal (C) or a *daf-6*(*e1377*) adult mutant (E). Arrow in (C), sensory cilium. Red arrowheads indicate subcortical electron dense material. Arrow in (E), bent cilium. Asterisk, bloated sheath glia channel. Note difference in magnification between (C) and (E). (F, H) Longitudinal section through the amphid primordium of a wild-type (F) or *daf-6*(*e1377*) (H) embryo at approximately 400 min of development. Asterisk, filaments. Arrow, basal body. (G, I) Cross-section through the amphid primordium of a wild-type (G) or *daf-6*(*e1377*) (I) embryo at approximately 420 min of development. Arrow, basal body. Asterisk, bloated channel. Note difference in magnification between (G) and (I). See also Figure S1.

To determine how such compartments form, we turned to the major sense organ of the nematode *Caenorhabditis elegans*, the amphid. *C. elegans* has two bilaterally symmetric amphids located in the head [11]. Each amphid consists of 12 sensory neurons, which mediate many of the behavioral responses of the animal, and two glial cells, the sheath and socket glia (Figure 1A, top). Amphid neurons are bipolar, projecting an axon into the nerve ring (the main neuropil of the animal) and extending a dendrite anteriorly to the tip of the nose. The two amphid glia also extend anterior processes collateral to the dendrites. At the nose tip, sheath and socket glia form discrete single-cell tubular channels joined by adherens junctions (Figure 1A bottom). The resulting two-cell channel compartment is open to the environment anteriorly and surrounds and isolates the ciliated endings of specific amphid sensory neurons. The socket portion of the channel is lined with cuticle and serves as a conduit for cilia to sample the animal's environment [11]. The sheath glial cell, however, is an active secretory cell [11], releasing extracellular matrix proteins, required for sensory neuron function, into the sheath glia channel [8].

Previous studies demonstrated that the morphogenesis of this compartment depends on the Patched-related gene *daf-6* [12]–[14], which acts within glia [14],[15]. Although the primary defects in *daf-6* mutants were not characterized, these studies demonstrated that glial compartment formation employs mechanisms shared with the genesis of other tubular structures in the animal, including the vulva and excretory system [14]. Similarly, the *C. elegans* Dispatched-related protein CHE-14 seems to play important roles in the formation of the amphid sensory compartment and other tubular organs [14],[16].

Here we demonstrate a primary function for *daf-6* in restricting sensory compartment size and show that the conserved MAP kinase LIT-1/NLK acts in counterbalance to DAF-6 to promote compartment expansion. Although LIT-1 is an important component of the Wnt signaling pathway in *C. elegans* [17], our studies argue against a role for Wnt in compartment size control. However, the previously characterized LIT-1 activator MOM-4/TAK1 is important for amphid sensory compartment morphogenesis. LIT-1 and MOM-4 co-localize to the amphid sensory compartment, and LIT-1 localization requires its highly conserved carboxy-terminal region. We demonstrate that this C-terminal domain physically interacts with actin and with the Wiskott-Aldrich syndrome protein (WASP), a regulator of actin polymerization [18]. Actin is highly enriched around the amphid pocket, and WASP appears to act in the same pathway as LIT-1 to influence compartment morphogenesis.

Our studies reveal two opposing activities, one mediated by DAF-6, the other by LIT-1, which, together with glial cytoskeletal proteins, drive sensory compartment morphogenesis.

## Results

### *daf-6*/Patched-Related Inhibits Amphid Sensory Channel Growth

The amphid sheath glial cell forms a compartment that surrounds the ciliated endings of amphid sensory neurons, constraining them into a tight bundle (Figure 1A–C). Within this bundle, 10 sensory cilia are stereotypically arranged in three successive columns containing 3, 4, and 3 cilia, respectively (Figure 1C; [11]). We previously reported the cloning and characterization of *daf-6*, a Patched-related gene required for amphid channel morphogenesis [14]. In *daf-6* mutant adults, the amphid channel is grossly enlarged, the socket and sheath glia channels are not continuous, and distal portions of sensory cilia are neither bundled nor exposed to the environment (Figure 1D and 1E).

At least two interpretations of this phenotype are possible: First, *daf-6* might act to open the sheath glia channel at its anterior end. Thus in *daf-6* mutants, the channel pocket would form but would remain sealed, and would continuously enlarge as matrix material is deposited. Second, *daf-6* might act to constrain the luminal diameter of the sheath glia channel. Thus, in *daf-6* mutants, the sheath and socket glia would properly align and form an open compartment, yet without lateral constraints on its size, the sheath channel would expand circumferentially. In this latter model, loss of the sheath-socket junction would be a later secondary defect.

To discriminate between these possibilities, we used electron microscopy (EM) to follow the development of amphid sensory compartments in wild-type and *daf-6*(*e1377*) mutant embryos. We used high-pressure freezing to fix embryos at several time points between 300 and 450 min post-fertilization, the time period during which the amphid is generated [19], collected serial sections, and assessed channel morphology.

By 380 min, sensory dendrites that have not yet formed cilia are evident in wild-type embryos. The tips of these dendrites are laterally ensheathed by the sheath glial cell, but the sheath cell also forms a cap blocking the anterior portion of the compartment and preventing access of neuronal processes to the socket (Figure S1).

By 400 min, a well-defined amphid primordium is formed in wild-type embryos (Figures 1F and S1). The sheath glia cap is gone and the open channel is continuous with the socket glia channel. At this stage, the socket channel is devoid of neuronal processes as dendritic tips have yet to extend cilia. Instead, a dense arrangement of filaments traverses the socket channel and forms a link between the tips of the sensory dendrites and the outside of the embryo (asterisk in Figure 1F). These filaments are consistent with an extracellular matrix proposed to anchor dendrites during retrograde extension [20]. Although cilia have not yet formed, structures resembling basal bodies (the initial sites of cilia construction) are visible at dendrite endings (arrow in Figure 1F).

In *daf-6* mutant embryos, the initial stages of amphid development are unperturbed (*n* = 3). By 400 min, the sheath and socket channels are aligned and open. Dendrites lacking cilia, but containing basal body-like structures, reside within the sheath channel, while filaments emanating from the dendrite tips and traversing the sheath and socket channels are seen (Figure 1H). However, only slightly later, at 420 min and before cilia have formed, bloating of the amphid sheath channel is apparent, and dendrites begin to unbundle (Figure 1I, compare to Figure 1G).

These studies indicate that *daf-6* is not required for aligning the sheath and socket channels or for opening the amphid sensory compartment. Rather, *daf-6* seems to function in restricting compartment diameter.

### Loss of *lit-1*/NLK Restores Amphid Sensory Compartment Morphology and Function to *daf-6* Mutants

The abnormal expansion of the amphid sensory compartment in *daf-6* mutants suggests that active processes promote compartment expansion and that these processes are balanced by *daf-6* activity during development. We surmised that mutations in genes promoting compartment expansion might, therefore, counteract the loss of *daf-6* and restore compartment size and function.

To identify such genes, we screened for mutants able to generate a normal compartment in the absence of *daf-6* function, taking advantage of an easily scored *daf-6* mutant defect: the inability to form dauer larvae. Dauer is an alternative developmental state induced by starvation and perception of high concentration of dauer pheromone. Dauer animals are highly resistant to environmental insults and can survive in the presence of 1% sodium-dodecylsulfate (SDS) [21]. *daf-6* mutants fail to become dauer larvae, presumably due to their sensory deficits [22], and are thus killed by exposure to SDS. We therefore randomly mutagenized animals homozygous for the strong loss-of-function *daf-6*(*e1377*) allele [14] using ethyl methanesulfonate (EMS), allowed F2 animals to starve, and treated them with SDS. Resistant animals could have suppressed the *daf-*6 amphid sensory compartment defects or could have constitutively activated a more downstream step in dauer formation. To distinguish between these mutant classes, we examined the ability of amphid sensory neurons to fill with dye provided in the medium. When exposed to a solution of the lipophilic dye DiI, wild-type animals readily take up the dye into exposed amphid neurons. *daf-6* animals fail to do so, presumably due to their defective amphid sensory compartments (Figure S2A–C) [13],[23].

**Figure 2:**
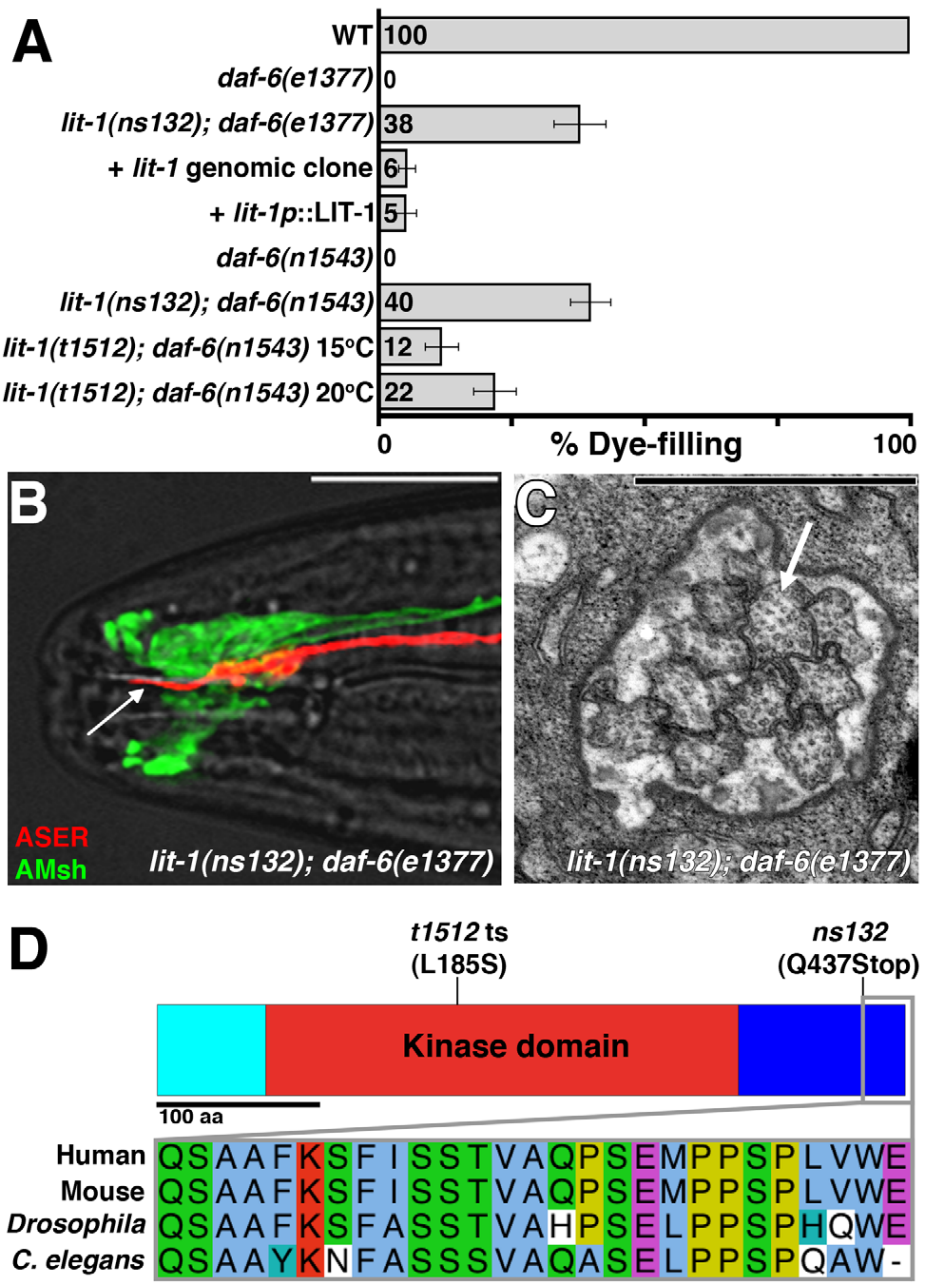
Loss of *lit-1* suppresses the loss of *daf-6.* (A) Dye-filling assay for indicated genotypes (*n*≥90). The *lit-1*(*t1512*) strain also contained the *unc-32*(*e189*) mutation. *unc-32*(*e189*) does not affect dye filling (unpublished data). Error bars, standard error of the mean (SEM). (B) The ASER neuron and the amphid sheath glia, visualized with mCherry (red) and GFP (green), respectively, in a *lit-1*(*ns132*); *daf-6*(*e1377*) animal (transgene *nsEx2761*). Arrow, ASER cilium. Left is anterior. Scale bar, 10 µm. (C) Electron micrograph of a cross-section through the amphid sheath channel of a *lit-1*(*ns132*); *daf-6*(*e1377*) adult animal. Arrow, cilium. Scale bar, 1 µm. (D) Top: Schematic of the LIT-1 protein. Light blue, non-conserved N-terminal domain. Red, conserved kinase domain. Dark blue, conserved C-terminal domain. Bottom: Alignment of the region truncated in *lit-1*(*ns132*) from different species. See also Figure S2.

From a screen of 60,000 mutagenized genomes we identified seven mutants that survived SDS treatment and that dye filled properly. We further characterized one of these *daf-6* suppressors, given the allele designation *ns132*. As shown in Figure 2A, approximately 40% of *ns132; daf-6*(*e1377*) animals are able to take up dye in at least one amphid. Likewise, the *ns132* allele was able to suppress amphid channel defects in another *daf-6* mutant, *n1543*, supporting the notion that *ns132* is a bypass suppressor (Figure 2A).

To further confirm the rescue of the *daf-6* amphid defects in *ns132*; *daf-6*(*e1377*) animals, we examined amphid sensory compartments using fluorescence microscopy. We found that cilia in these double mutants projected through a compartment of normal appearance (Figure 2B, compare to Figure 1D). In addition, *ns132*; *daf-6*(*e1377*) individuals that displayed normal dye filling in one of the two amphids had one amphid channel that resembled a wild-type channel by EM serial reconstruction (Figure 2C; *n* = 3). Interestingly, even in rescued amphids, cilia packing was more variable compared to the regular 3∶4∶3 packing observed in wild-type animals, and the amphid sensory compartment was somewhat wider than normal (Figure 2C, compare to Figure 1C), perhaps reflecting a partial suppression of the *daf-6* defects.

We used single nucleotide polymorphism (SNP) mapping and transgenic rescue methods (Figure S2D) to identify the gene defective in *ns132* animals as *lit-1*. *lit-1* encodes a Ser/Thr MAP kinase that is highly conserved from *C. elegans* to mammals. Supporting this assignment, a genomic region containing *lit-1* restored dye-filling defects to *ns132*; *daf-6*(*e1377*) animals (Figures 2A and S2E), as did a transgene in which the *lit-1* promoter region (2.5 kb upstream of the *lit-1* start codon) drives the *lit-1* cDNA (Figure 2A). Furthermore, a temperature-sensitive mutation in *lit-1*, *t1512*, also suppressed the dye-filling defects of *daf-6*(*n1543*) mutants (Figure 2A). Finally, we found that animals containing the *ns132* allele have a C-to-T mutation in the coding region of *lit-1*, converting codon 437, encoding glutamine, to a stop codon. This mutation is predicted to result in a truncated LIT-1 protein (Figure 2D) lacking the last 26 amino acids of the highly conserved carboxy-terminal (C-terminal) domain.

### LIT-1 Functions in Amphid Glia During Compartment Formation

**Figure 3:**
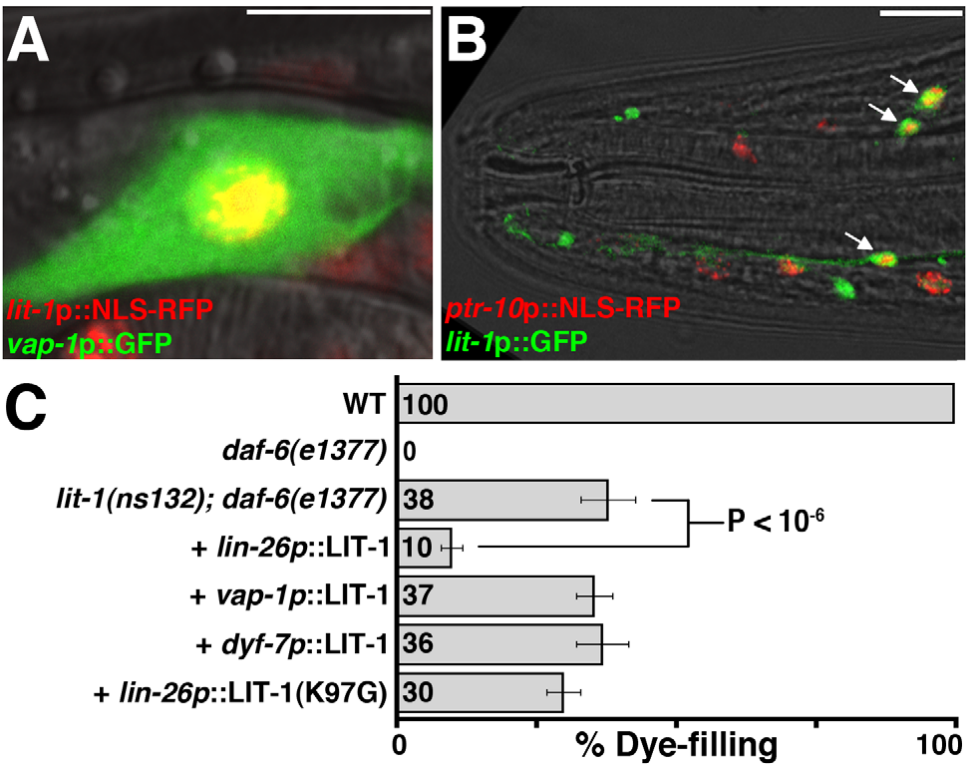
Suppression of *daf-6* mutations requires loss of *lit-1* in glia. (A) Image of an amphid sheath glial cell body expressing *lit-1*p::NLS-RFP (red; in nucleus) and *vap-1*p::GFP (green) (transgene *nsEx2308*). Yellow, overlapping expression. Left is anterior. Scale bar, 10 µm. (B) Image of an adult (head) expressing *lit-1*p::GFP (green) and *ptr-10*p::NLS-RFP (red) (transgene *nsEx2159*). Arrows, cells with overlapping expression. Left is anterior. Scale bar, 10 µm. (C) Dye-filling assay for indicated genotypes (*n*≥90). None of the transgenes had an effect on the dye filling of wild type animals (*n*>100, unpublished data). Error bars, SEM. *p* value calculated using Chi-squared test.

To determine in which cells *lit-1* functions to regulate compartment development, we first examined its expression pattern by generating animals harboring a transgene in which the *lit-1* promoter drives expression of a nuclearly localized dsRed fluorescent protein (NLS-RFP). We found that *lit-1* is expressed in amphid sheath glia (Figure 3A), among other cells. In addition, the expression pattern of this reporter partially overlaps with that of *ptr-10* (Figure 3B), a gene expressed in ensheathing glia of other sensory organs [24], suggesting that *lit-1* could act in compartment formation in other *C. elegans* sensory structures as well.

Next, we pursued cell-specific rescue experiments to determine in which cells *lit-1* can act to regulate compartment morphogenesis. We generated *lit-1*(*ns132*); *daf-6*(*e1377*) animals containing a transgene in which a *lin-26* promoter fragment drives expression of the *lit-1* cDNA in glia, but not neurons, of embryos at the time of amphid sensory compartment formation [25]. We found that transgenic animals were rescued (Figure 3C), supporting the notion that *lit-1* can act in glia to regulate compartment morphology. Importantly, expression of the *lit-1* cDNA in amphid sensory neurons during the time of amphid morphogenesis (using the *dyf-7* promoter; [20]) failed to rescue *lit-1*(*ns132*); *daf-6*(*e1377*) animals (Figure 3C).

To determine whether *lit-1* can control amphid sensory compartment structure after compartment formation is complete, we examined *lit-1*(*ns132*); *daf-6*(*e1377*) animals expressing the *lit-1* cDNA under the control of the sheath glia-specific *vap-1* promoter. *vap-1* expression begins in late embryos [14], after the compartment has formed. We found that these transgenic animals were not rescued (Figure 3C), supporting the conclusion that *lit-1* is required within amphid sheath glia at the time of amphid morphogenesis to influence compartment formation.

Finally, to ascertain whether the kinase activity of LIT-1 is required, we generated a mutant *lit-1* cDNA that disrupts the ATP binding domain (VALKK to VALGK) and which has been shown to eliminate LIT-1 kinase activity in vitro [17]. *lit-1*(*ns132*); *daf-6*(*e1377*) animals carrying a *lin-26* promoter::LIT-1(K97G) cDNA transgene still displayed 30% dye filling, similar to controls, suggesting that LIT-1 kinase activity is indeed required for glial compartment morphogenesis (Figure 3C). None of the transgenes used in Figure 3C had an effect on the dye filling of wild-type animals (*n*>100).

### *lit-1* Promotes Amphid Sensory Compartment Expansion

**Figure 4:**
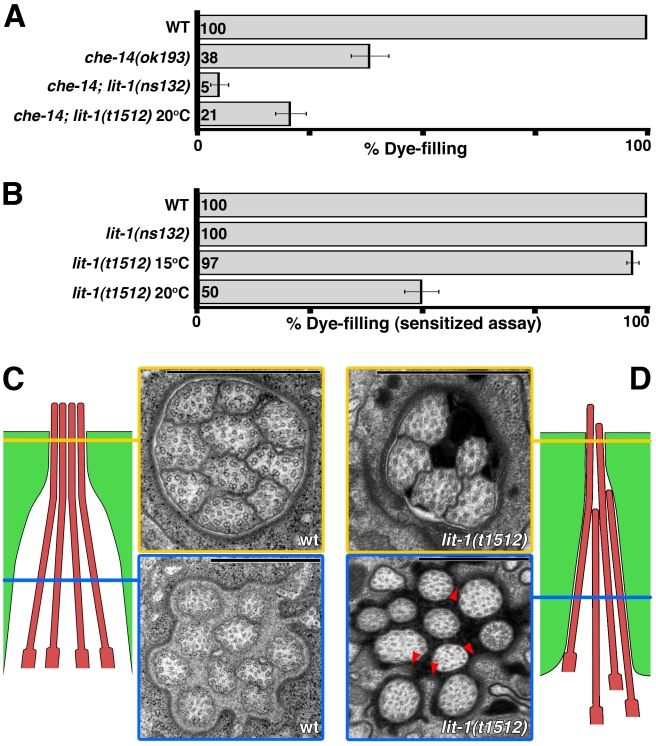
LIT-1 is required for amphid sensory compartment morphogenesis. (A, B) Dye filling in animals carrying the indicated mutations (*n*≥100). Error bars, SEM. In (B) a sensitized dye-filling assay was used (see Experimental Procedures). (C) Left: Schematic of the arrangement of the cilia (red) and the sheath glial channel (green) in a wild-type adult animal. Not all cilia are depicted. Right: electron micrograph of cross-sections of the amphid channel. Section outlined in yellow is just below the socket-sheath junction; blue outlined section is approximately one micron posterior. Scale bars, 1 µm. (D) Same as in (C), but for a dye-filling defective *lit-1*(*t1512*) adult animal. The panel arrangement is a reflection of the one in (C). Arrowheads, tight ensheathment of individual cilia by the sheath glia. Scale bars, 1 µm.

Since *daf-6* normally acts to restrict amphid sensory compartment expansion, the observation that *lit-1* mutations suppress *daf-6* suggests that *lit-1* may normally promote compartment growth. Consistent with this idea, the *lit-1*(*ns132*) allele enhances the dye-filling defects of *che-14*(*ok193*) mutants (Figure 4A). CHE-14 protein is similar to the *Drosophila* and mammalian protein Dispatched, and is important for apical secretion and amphid sensory compartment morphogenesis [16], suggesting a role in lumen expansion. The enhancement of *che-14* defects by *lit-1*(*ns132*) suggests that both genes may be involved in this process.

To further test the idea that *lit-1* promotes compartment expansion, we examined *lit-1*(*ns132*) single mutants for dye-filling abnormalities; however, no defects were observed (Figure 4B), suggesting that amphid morphology in these animals may be normal. However, two observations suggest that *ns132* is a weak allele of *lit-1*. First, the *ns132* lesion truncates only 26 amino acids from the C-terminus of the LIT-1 protein and leaves the kinase domain intact (Figure 2D). Second, null alleles of *lit-1* are embryonic lethal [17],[26], whereas *ns132* mutants are fully viable.

To examine the consequences of more severe defects in *lit-1* function, we turned to animals homozygous for the *lit-1*(*t1512*) temperature-sensitive allele. *lit-1*(*t1512*ts) animals grow nearly normally at 15°C, but exhibit early embryonic lethality at 25°C [26]. At 20°C, some *lit-1*(*t1512*ts) embryos escape lethality and grow to adulthood. We reasoned that in some of these escapers, LIT-1 activity could be low enough to allow us to discern defects in amphid morphogenesis. Indeed, as shown in Figure 4B, nearly 50% of *lit-1*(*t1512*ts) adults grown at 20°C exhibit defects in a sensitized amphid dye-filling assay (this assay was developed to detect weak defects in dye filling; see Experimental Procedures). These results suggest that amphid structure, and perhaps compartment morphogenesis, has been perturbed in these mutants.

To assess whether compartment morphology is indeed perturbed, we performed serial-section EM on dye-filling defective adult *lit-1*(*t1512*ts) animals raised at 20°C (*n* = 3). Whereas in wild-type animals a cross-section through the sheath channel immediately posterior to the socket-sheath boundary (yellow line in Figure 4C) reveals the stereotypical 3∶4∶3 arrangement of the 10 channel cilia, in *lit-1*(*t1512*ts) mutants (Figure 4D), the amphid sensory compartment has a smaller diameter and contains fewer cilia. Fewer cilia are also found in the socket channel in *lit-1*(*t1512*ts) animals (unpublished data). Furthermore, in wild-type animals, cross-sections roughly 1 µm posterior to the sheath-socket junction (blue line in Figure 4C) reveal a less packed arrangement of cilia that are loosely surrounded by the sheath glia membrane; by contrast, in *lit-1*(*t1512*ts) animals the sheath glia is tightly wrapped around individual cilia (arrowheads in Figure 4D), consistent with the idea that compartment diameter is reduced. Importantly, despite the posterior displacement of some cilia in *lit-1*(*t1512*ts) animals, the total number of cilia is normal (blue section in Figure 4D).

Taken together, the *che-14*, dye-filling, and EM studies suggest that *lit-1* opposes *daf-6* by promoting channel expansion during amphid morphogenesis.

### Mutation of the MAP Kinase Kinase Kinase *mom-4*/TAK1 Also Suppresses the Compartment Defects of *daf-6* Mutants

**Figure 5:**
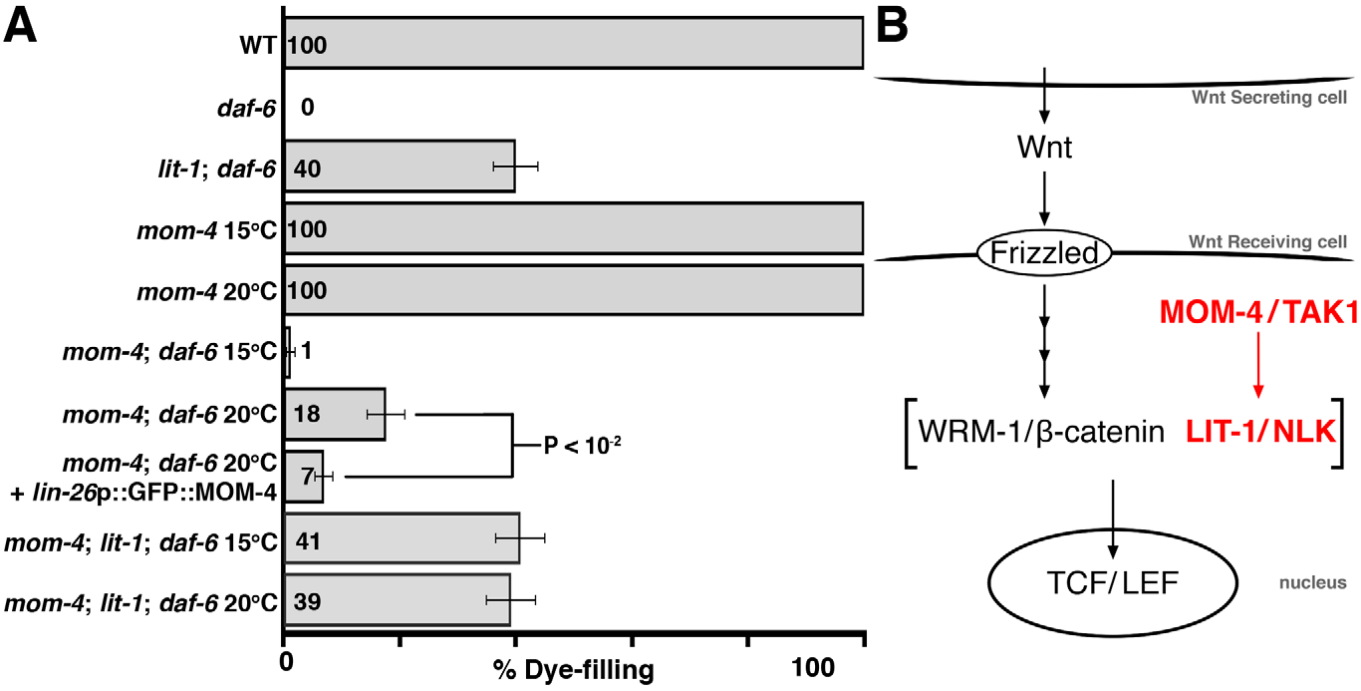
*mom-4*/TAK1 mutations suppress the loss of *daf-6.* (A) Dye filling in animals of the indicated genotypes (*n*≥90). The alleles used are: *daf-6*(*n1543*), *lit-1*(*ns132*), *mom-4*(*ne1539*). *daf-6* is marked with *unc-3*(*e151*) in all strains except for *mom-4; daf-6*. *unc-3*(*e151*) does not affect dye filling (unpublished data). Error bars, SEM. *p* value calculated using Chi-squared test. (B) Schematic of Wnt signaling during endoderm specification in *C. elegans*. In contrast to the LIT-1 MAPK module (red), Wnt signaling does not appear to be involved in amphid sheath channel formation (see text and Table S1).

The kinase activity of LIT-1 was previously shown to depend on MOM-4/TAK1, a MAP kinase kinase kinase. MOM-4 increases LIT-1 kinase activity in vitro and mutations in *mom-4* interact genetically with mutations in *lit-1* during anterior/posterior polarity establishment in early embryos [27]. We therefore tested whether mutations in *mom-4* could also suppress the dye-filling defects of *daf-6* mutants. While complete loss of *mom-4*, like loss of *lit-1*, leads to early embryonic lethality, some animals homozygous for a temperature-sensitive allele of *mom-4*, *ne1539*ts, can escape lethality. We found that whereas only 1% of *mom-4*(*ne1539*ts); *daf-6*(*e1377*) double-mutant escapers grown at 15°C exhibit suppression of the *daf-6* dye-filling defect, 18% of surviving animals grown at 20°C can take up dye (*p*<10^−6^, Chi-squared test; Figure 5A). This observation suggests that *mom-4* acts similarly to *lit-1* in compartment expansion.

To test whether *mom-4*, like *lit-1*, acts within glia to regulate amphid morphogenesis, we constructed *mom-4*(*ne1539*ts); *daf-6*(*e1377*) double mutants expressing a *lin-26* promoter::GFP::*mom-4* cDNA transgene. When these animals were grown at 20°C, only 7% filled with dye (Figure 5A), consistent with the hypothesis that *mom-4* acts within glia during early amphid morphogenesis, similar to *lit-1*.

To assess whether *mom-4* and *lit-1* function in the same pathway to promote channel expansion, we examined dye filling in *daf-6* mutants that were also homozygous for both *lit-1*(*ns132*) and *mom-4*(*ne1539*ts) alleles. We found that the *mom-4*; *lit-1*; *daf-6* triple mutant is viable at both 15°C and 20°C and is not suppressed to a greater extent than *lit-1*; *daf-6* double mutants at either temperature (Figure 5A). This result is consistent with the idea that *lit-1* and *mom-4* function in the same pathway to control channel expansion, similar to their established roles in embryonic cell polarity.

The roles of *lit-1* and *mom-4* in Wnt signaling in *C. elegans* have been extensively studied [28],[29]. In this context, MOM-4 activates LIT-1, which then forms a complex with the β-catenin WRM-1. The LIT-1/WRM-1 complex phosphorylates the *C. elegans* TCF/LEF transcription factor POP-1, resulting in reduction (but not elimination) of POP-1 nuclear levels and activation of transcription (Figure 5B) [17],[27],[30],[31]. We therefore examined animals containing mutations in Wnt signaling components for defects in dye filling, or for suppression of the *daf-6* dye-filling defects. Surprisingly, mutations in Wnt-encoding genes, the *C. elegans* Wntless homolog *mig-14*, required for Wnt protein secretion, Wnt receptors, β-catenins, or *pop-1*/TCF/LEF, the main LIT-1 target in the Wnt signaling pathway, have no effect on dye filling and show no, or minimal, suppression of *daf-6* (Table S1).

Although we cannot eliminate the possibility that multiple redundant Wnt pathways contribute to channel formation and that these operate through LIT-1 targets other than POP-1, the most parsimonious interpretation of our data is that the MOM-4/LIT-1 kinase module operates independently of Wnt signaling to promote expansion of the amphid glial compartment.

### LIT-1 and MOM-4 Proteins Localize to the Amphid Sensory Compartment

**Figure 6:**
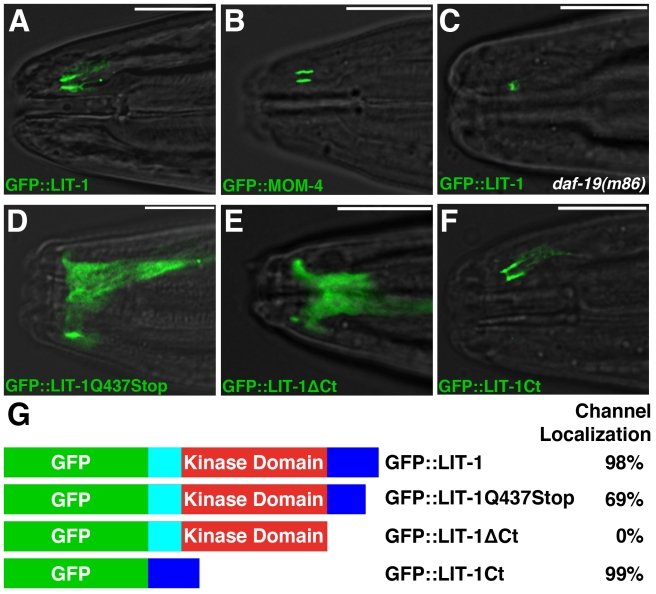
LIT-1 and MOM-4 localize to the amphid sensory compartment. (A–F) Images of adult animals expressing the indicated GFP fusion proteins. Animals are otherwise wild-type except in (C). *daf-19*(*m86*) animals also carried the *daf-16*(*mu86*) allele to prevent dauer entry. The *T02B11.3* amphid sheath promoter [32] was used to drive all constructs. Transgenes depicted: *nsEx2606* (A), *nsEx2840* (B), *nsEx2829* (C), *nsEx2609* (D), *nsEx2747* (E), and *nsEx2626* (F). Anterior is to the left. Scale bars, 10 µm. (G) Quantification of channel localization of indicated LIT-1 protein fusions (*n*≥100). See also Figure S3.

To determine where within the amphid sheath glia LIT-1 and MOM-4 are localized, we generated animals expressing either a rescuing GFP::MOM-4 or a rescuing GFP::LIT-1 fusion protein within amphid sheath glia using the *T02B11.3* amphid sheath promoter [32]. Strikingly, we found that both fusion proteins were tightly associated with the amphid sensory compartment (Figure 6A and 6B).

To determine whether LIT-1 localization requires functional *mom-4*, we examined localization of the GFP::LIT-1 fusion protein in *mom-4*(*ne1539*ts) single mutants at 20°C. GFP::LIT-1 was properly localized in all animals we observed (*n* = 44), suggesting that LIT-1 localizes to the sheath channel independently of its regulator.

The DAF-6 protein is mislocalized in animals lacking neuronal cilia, accumulating only at the sheath-socket junction rather than along the length of the sheath glia channel [14]. To examine whether LIT-1 also requires cilia to properly localize, we examined animals harboring a loss-of-function mutation in *daf-19*, which encodes a transcription factor required for ciliogenesis. Our previous EM studies demonstrated that, despite minor defects, a channel of normal length is generated in these mutants [14]. As shown in Figure 6C, in *daf-19* mutants, LIT-1 no longer lines the entire channel, but is restricted to its anterior aspect. Thus, neuronal signals are required for LIT-1 glial localization.

### The C-Terminus of LIT-1 Is Necessary and Sufficient for Amphid Sensory Compartment Localization

The channel localization of LIT-1 raised the possibility that in *lit-1*(*ns132*) mutants, LIT-1 localization might be disrupted. To test this, we expressed GFP-tagged LIT-1(Q437Stop) (the mutation corresponding to *ns132*) in wild-type animals and examined its localization. While GFP::LIT-1 reproducibly lines the amphid sensory compartment, GFP::LIT-1(Q437Stop) fails to localize in about one-third of animals and is instead diffusely distributed throughout the cell (Figure 6D and 6G). This result suggests that the highly conserved C-terminal region of LIT-1 may be required for compartment localization. In addition, the fraction of animals in which GFP::LIT-1(Q437Stop) is mislocalized (31%, Figure 6G) mirrors the fraction of *daf-6* mutants suppressed by the *lit-1*(*ns132*) allele (Figure 2A), raising the possibility that mislocalization may account for the suppression we observed.

The observation that GFP::LIT-1(Q437Stop) still localizes to the amphid channel in some animals raised the possibility that the C-terminal 26 amino acids may represent only a portion of the full targeting domain. To test this idea, we generated animals expressing a GFP::LIT-1ΔCt fusion protein in which all sequences downstream of the kinase domain are deleted. We found that in these animals LIT-1 never accumulated at the amphid sensory compartment, and was diffusely distributed throughout the cell (Figure 6E and 6G), demonstrating that the C-terminal domain is necessary for LIT-1 compartment localization.

To determine whether the C-terminal domain of LIT-1 is sufficient for channel localization, we generated animals expressing a GFP::LIT-1 C-terminal domain fusion protein. Remarkably, we found that this fusion protein accumulated at the amphid sensory compartment in a pattern identical to that of full-length LIT-1 (Figure 6F and 6G).

Previous work showed that LIT-1 also localizes to the cell nucleus [30],[33],[34], and we found this to be the case for amphid sheath glia as well (Figure S3). However, disruption of the C-terminal domain of LIT-1 does not result in its exclusion from the nucleus (Figure S3), suggesting that nuclear functions of LIT-1 may not be abrogated in *lit-1*(*ns132*) mutants.

Although the C-terminal domain of LIT-1 is highly conserved from *C. elegans* to mammals, its function is not well studied. Our studies demonstrate that this domain is both necessary and sufficient for LIT-1 localization to the amphid sensory compartment, and suggest that proper localization is important for LIT-1 function in compartment formation.

### ACT-4 Interacts with the C-terminal Domain of LIT-1 and Is Enriched around the Amphid Sensory Compartment

Because of the importance of the LIT-1 C-terminal domain in compartment localization, we used this domain as bait in a yeast two-hybrid screen with the aim of identifying proteins that interact with LIT-1.

**Figure 7:**
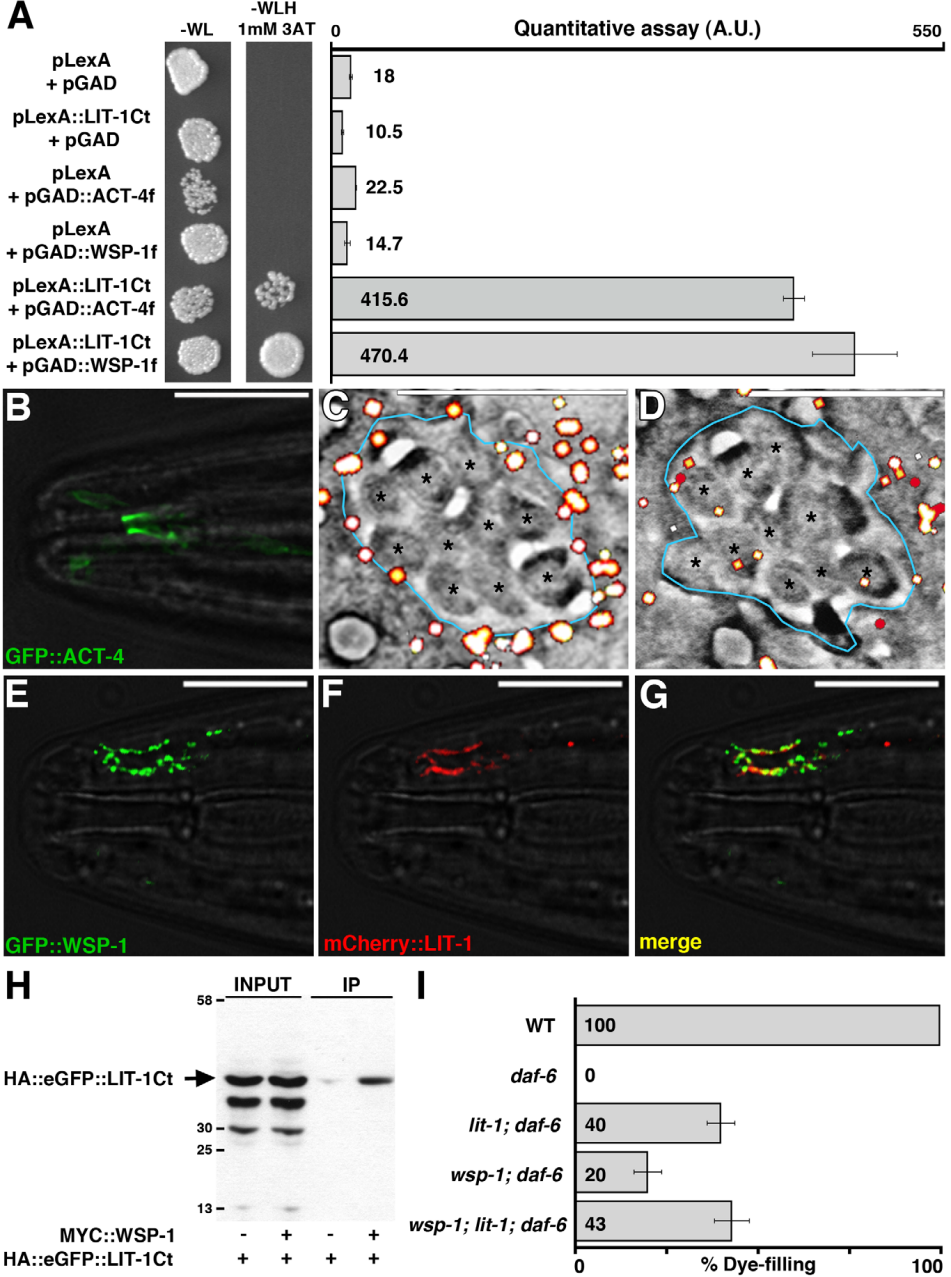
The actin cytoskeleton is involved in amphid sensory compartment morphogenesis. (A) Growth assay (left) and quantitative β-galactosidase enzymatic activity assay (right) demonstrating the interaction between LexA fused to the LIT-1 carboxy-terminal domain and GAD fused to fragments of ACT-4 or WSP-1. Error bars, standard deviation. f, fragment. −WL, medium without Tryptophan and Leucine. –WLH, medium without Tryptophan, Leucine, and Histidine. 3AT, 3-amino-1,2,4-triazole. A.U., arbitrary units. (B) Amphid channel localization of GFP::ACT-4 (transgene *nsEx2876*). Anterior is to the left. Scale bar, 10 µm. (C, D) fEM (see Experimental Procedures) of a cross-section through the amphid channel (blue trace) just below the socket-sheath junction (C) or 2 µm posterior (D). White puncta indicate mEos::ACT-4 localization. Transgene used *nsEx2970*. Asterisks, cilia. Scale bars, 1 µm. (E–G) Co-localization of GFP::WSP-1 and mCherry::LIT-1 at the amphid sensory compartment (transgene *nsEx3245*). The *T02B11.3* amphid sheath promoter [32] was used to drive all constructs. Anterior is to the left. Scale bars, 10 µm. (H) The carboxy-terminal domain of LIT-1 co-immunoprecipitates with WSP-1. *Drosophila* S2 cells were transfected with HA::eGFP::LIT-1Ct and with or without MYC::WSP-1. Cell lysates were immunoprecipitated using anti-MYC-conjugated agarose beads and analyzed by anti-HA immunoblot. (I) Dye filling in animals of the indicated genotypes (*n*≥90). The alleles used are: *daf-6*(*n1543*), *lit-1*(*ns132*), *wsp-1*(*gm324*). *daf-6* is marked with *unc-3*(*e151*) in all strains. *unc-3*(*e151*) does not affect dye filling (unpublished data). Error bars, SEM. See also Table S2.

From a screen of approximately 10^6^ clones, we identified 26 positive clones (Table S2, Figure 7A). While some clones were isolated multiple times, others were found only once, suggesting that our screen was not saturated. We were intrigued that 4 of the 26 interacting clones identified encoded the *C. elegans* actin protein ACT-4. EM studies of the amphid sheath glia channel had previously shown that the channel is lined by an electron dense subcortical layer (red arrowheads in Figure 1C) [13]. A similar layer can be seen in other highly secreting cells such as pancreatic acinar cells and adrenal chromaffin cells. In these cells, this electron dense layer has been demonstrated to be enriched in actin [35],[36].

To determine whether ACT-4 might be part of the electron-dense subcortical layer near the amphid sensory compartment, we examined animals expressing a GFP::ACT-4 fusion protein in amphid sheath glia. Strikingly, we found that although GFP::ACT-4 was seen throughout the cell, it was highly enriched at the amphid sensory compartment (Figure 7B). We wondered whether other actin proteins also accumulate at the channel and, therefore, generated animals expressing a protein fusion of GFP to ACT-1. Again, we found increased channel localization (unpublished data), suggesting that actin filaments may be components of the subcortical density.

To examine the localization pattern of ACT-4 at higher resolution, we used scanning EM coupled with photo-activated localization microscopy (PALM). In this method, serial sections are imaged by scanning EM and using single-molecule fluorescence of mEos::ACT-4 [37]. Images are then superimposed, using fiduciary markers (fluorescent gold beads), to reveal the subcellular localization of fluorescent proteins. As shown in Figure 7C, at the anterior portion of the amphid channel, where an electron dense subcortical region has been described, mEos::ACT-4 is localized near the sensory compartment membrane (blue trace). mEos::ACT-4 does not localize to the sensory compartment in more posterior sections (Figure 7D, 2 µm posterior to 7C), which should lack the subcortical electron density. These observations support the notion that actin is intimately associated with the glial sensory compartment and that the subcortical density may be composed at least in part of actin.

We also found that GFP::ACT-4 was properly localized in *lit-1*(*ns132*) mutants (*n* = 50), suggesting that actin accumulates around the sensory compartment independently of *lit-1*, and consistent with the possibility that actin may recruit LIT-1. To test this possibility we tried to disturb GFP::ACT-4 localization by treating the animals with an inhibitor of actin polymerization, cytochalasin D. After a 2 h incubation with 1 mM of the drug, the cell bodies of the sheath glia assumed a rounded morphology, indicative of breakdown of the actin cytoskeleton. However, the sensory compartment localization of neither GFP::ACT-4 nor GFP::LIT-1 was disturbed (unpublished data). This result suggests that the subcortical actin around the amphid channel could be part of a stable structure with a lower turnover rate than the rest of the actin cytoskeleton.

Similarly, LIT-1, MOM-4, and ACT-4 all localized to the sensory compartment in *daf-6*(*n1543*) mutants (Figure S4), suggesting that DAF-6 is not involved in recruiting these proteins.

### The Actin Regulator WASP Binds LIT-1 and Is Required for Sensory Compartment Expansion in *daf-6* Mutants

In addition to actin, our two-hybrid studies suggested that the LIT-1 C-terminal domain can also bind to the proline-rich region of WSP-1, the *C. elegans* homolog of the Wiskott-Aldrich Syndrome Protein (WASP) (Table S2, Figure 7A). Furthermore, we could immunoprecipitate the LIT-1 C-terminal domain using WSP-1 from cultured *Drosophila* S2 cells co-expressing both proteins (Figure 7H), suggesting that LIT-1 and WSP-1 can interact. Although GFP::WSP-1 expressed in amphid sheath glia is diffusely localized (unpublished data), co-expression with mCherry::LIT-1 revealed partial co-localization (Figure 7E–G), supporting the notion that LIT-1 and WSP-1 may interact in vivo.

To determine whether *wsp-1* plays a role in amphid morphogenesis, we examined *wsp-1*(*gm324*) mutants, which, unlike actin mutants, are viable [38]. We did not find any defects in dye filling in the single mutant. However, *wsp-1*(*gm324*) suppresses the *daf-6*(*n1543*) dye-filling defects (Figure 7I). Furthermore, *daf-6* mutants homozygous for both *lit-1*(*ns132*) and *wsp-1*(*gm324*) were as dye-filling defective as *lit-1*(*ns132*); *daf-6*(*n1543*) mutants alone, consistent with the hypothesis that LIT-1 and WSP-1 act in the same pathway.

Interestingly, we found that overexpression of a GFP::LIT-1 fusion protein results in abnormal glial morphology (Figure S5B, compare to Figure S5A) and distorted sensory compartment morphology (Figure S5C, compare to Figure 6A). This result, together with the genetic and physical interactions between LIT-1 and actin and LIT-1 and WASP, are consistent with the possibility that LIT-1 facilitates glial morphogenesis by regulating actin dynamics.

## Discussion

### *lit-1* Regulates the Morphogenesis of a Subcellular Structure

LIT-1 is the *C. elegans* homolog of Nemo-like kinase (NLK) [39], a Serine/Threonine kinase originally described in *Drosophila* [40]. In *C. elegans*, *lit-1* (loss of intestine) was first identified for its role in endoderm specification during early embryogenesis [26]. Subsequent work established *lit-1* as a component of the Wnt/β-catenin asymmetry pathway that directs many cell fate decisions in *C. elegans* [28],[29]. NLK also plays roles in control of the Wnt [41],[42], TGFβ [43], and Notch [44] signaling pathways in vertebrates.

Although LIT-1/NLK has been implicated in cell fate determination, we identified *lit-1* mutations as suppressors of lesions in *daf-6*, a gene that affects morphogenesis of the amphid glial sensory compartment, but not glial cell fate. Indeed, *lit-1* single mutants seem to have well-specified amphid components. Furthermore, despite an established connection between *lit-1* and the Wnt/β-catenin asymmetry pathway (a major regulator of cell fate decisions in *C. elegans*), we found no evidence linking Wnt signaling to amphid morphogenesis (Table S1). These observations are consistent with the idea that the role of *lit-1* in sensory organ morphogenesis does not involve cell fate decisions, but instead reflects a novel function in cellular morphogenesis.

Within the context of cell fate decisions, LIT-1/NLK often acts by impinging upon the activity of nuclear transcription factors [30],[43],[44]. It is unclear whether the role of *lit-1* in sensory organ morphogenesis might also involve transcriptional regulation. The C-terminal domain of LIT-1 is required for its role in amphid morphogenesis and for its amphid channel localization, but it is not essential for the ability of LIT-1 to enter the nucleus. This suggests that LIT-1 may exert its primary influence on channel morphogenesis at the channel itself. However, LIT-1 C-terminus can interact not only with cytoskeletal proteins (actin and WASP) but also with the transcription factors ZTF-16 and MEP-1 (Table S2). Thus, while it is likely that sensory compartment localization is important for LIT-1 function, we cannot rule out the possibility that LIT-1 has independent relevant functions in the nucleus.

### Opposing Activities of *lit-1* and *daf-6* Direct Sensory Compartment Morphogenesis

Our results suggest that *daf-6* and *lit-1* direct the morphogenesis of the sheath glia sensory compartment by exerting opposing influences. In *daf-6* mutants, neurons and glia form an amphid primordium in which all components are initially linked and aligned; however, the sensory compartment expands abnormally. Conversely, in *lit-1* mutants, the sensory compartment is too narrow. Mutations in *lit-1* can correct for the loss of *daf-6*; thus, *lit-1*; *daf-6* double mutants have relatively normal glial channels. A situation that mimics *lit-1*; *daf-6* double mutants arises in animals with mutations in genes controlling neuronal cilia development. In these animals, channel localization of LIT-1, as well as DAF-6, is perturbed. Consistent with the *lit-1*; *daf-6* phenotype, channel formation is only mildly defective in these mutants [14].

The observation that *lit-1* loss-of-function mutations suppress *daf-6* null alleles argues that *lit-1* cannot function solely upstream of *daf-6* in a linear pathway leading to channel formation. Our data, however, are consistent with the possibility that *daf-6* functions upstream of *lit-1* to inhibit *lit-1* activity. Alternatively, *lit-1* and *daf-6* may act in parallel. Our studies do not currently allow us to distinguish between these models.

### Vesicles, the Actin Cytoskeleton, and Sensory Compartment Morphogenesis

How might DAF-6 restrict the size of glial sensory compartments? Electron micrographs of the *C. elegans* amphid reveal the presence of highly organized Golgi stacks near the amphid channel. These images also show vesicles, containing extracellular matrix, that appear to be released by the sheath glia into the channel (Figure 1A) [11]. These studies suggest that vesicular secretion may play a role in channel morphogenesis. Interestingly, DAF-6 is related to Patched, a protein implicated in endocytosis of the Hedgehog ligand, and the *C. elegans* Patched gene *ptc-1* is proposed to regulate vesicle dynamics during germ-cell cytokinesis [45]. Furthermore, DAF-6 can be seen in punctate structures, which may be vesicles [14], and DAF-6 and CHE-14/Dispatched function together in tubulogenesis [14],[16], a process hypothesized to require specialized vesicular transport. Together these observations raise the possibility that DAF-6 may restrict amphid sensory compartment expansion by regulating vesicle dynamics in the sheath glia [14].

If indeed DAF-6 controls membrane dynamics, it is possible that LIT-1, which localizes to and functions at the sheath glia channel, also interfaces with such processes. How might LIT-1 localize to the glial sensory compartment and control vesicle dynamics? Previous studies suggest that cortical localization of LIT-1 requires it to stably interact with WRM-1/β-catenin [33],[34]. In the sheath glia, however, we found that *wrm-1* is not required for sensory compartment morphogenesis or for LIT-1 localization and that LIT-1 and WRM-1 do not co-localize to the amphid sensory compartment (unpublished data). Instead, we found that LIT-1 physically interacts with actin and that actin is highly enriched around the amphid sensory compartment. Thus, actin might serve as a docking site for LIT-1. The interaction between LIT-1 and actin may not be passive. Indeed, we showed that LIT-1 also binds to WASP, and mutations in *wsp-1/*WASP suppress *daf-6* similarly to mutations in *lit-1*. Furthermore, WASP activity is stimulated by phosphorylation of Serines 483 and 484 [46], suggesting that LIT-1, a Ser/Thr kinase, could activate WASP to promote actin remodeling.

Remodeling of the cortical actin cytoskeleton plays important roles in several aspects of membrane dynamics [47]. For example, WASP-dependent actin polymerization has a well-established role in promoting vesicle assembly during clathrin-mediated endocytosis [48]. Recent work has demonstrated positive roles for actin polymerization in exocytosis as well [49],[50]. In pancreatic acinar cells, secretory granules become coated with actin prior to membrane fusion [51], and in neuroendocrine cells, actin polymerization driven by WASP stimulates secretion [52]. During *Drosophila* myoblast fusion, actin polymerization, dependent on WASP and WASP interacting protein (WIP), is required for targeted exocytosis of prefusion vesicles [53], and antibodies against WASP inhibit fusion of purified yeast vacuoles [54]. An attractive possibility, therefore, is that LIT-1 might regulate sensory compartment morphogenesis by altering vesicle trafficking through WASP-dependent actin polymerization.

Glial ensheathment is a feature of many animal sensory organs and synapses, and LIT-1 and WASP are highly conserved, suggesting that our studies may be broadly relevant. Interestingly, LIT-1 was recently shown to be required for cell invasion through basement membranes in *C. elegans* and in metastatic carcinoma cells [55], processes that require extensive remodeling of the actin cytoskeleton. Our results may, thus, represent a general mechanism for regulating cell shape changes using localized interactions of LIT-1/NLK with cytoskeletal proteins.

## Materials and Methods

### Strains, Plasmid Construction, and *lit-1* Mapping and Cloning

See Supporting Information.

### Dye-Filling Assay

Animals were washed off NGM plates with M9 buffer, resuspended in a solution of 10 µg/mL of DiI (1,1′-dioctadecyl-3,3,3′,3′-tetramethylindocarbocyanine perchlorate) (Invitrogen D282), and rotated in the dark for 1.5 h at room temperature. Animals were then transferred to a fresh NGM plate, anaesthetized with 20 mM sodium azide, and observed using a dissecting microscope equipped with epifluorescence. Animals in which none of the amphid neurons filled with dye were scored as dye-filling defective (Dyf). For the sensitized dye-filling assay, 1 µg/mL of DiI was used, and the incubation time was 15 min. Animals were scored as dye filling defective (Dyf) if either one or two amphids failed to fill.

### Transmission Electron Microscopy and Fluorescence Electron Microscopy (fEM)

See Supporting Information and [37].

### Fluorescence Microscopy and Image Analysis

Images were acquired using a DeltaVision Image Restoration Microscope (Applied Precision) equipped with a 60×/NA 1.42 Plan Apo N oil immersion objective (Olympus) and a Photometrics CoolSnap camera (Roper Scientific), or an Upright Axioplan LSM 510 laser scanning confocal microscope (Zeiss) equipped with a C-Apochromat 40×/NA 1.2 objective. Acquisition, deconvolution, and analysis of images from the DeltaVision system were performed with Softworx (Applied Precision); images from the confocal microscope were acquired and analyzed using LSM 510 (Zeiss).

### Yeast Two-Hybrid Screen

LexA::LIT-1Ct was used as bait in a Y2H screen using the DUALHhybrid kit (Dualsystems Biotech) in conjunction with the *C. elegans* Y2H cDNA library (Dualsystems Biotech), as described by the manufacturer. For the growth assay, cultures growing on Synthetic Complete Dextrose –Tryptophan, –Leucine (SCD –WL) plates were resuspended in water to OD_660_ = 0.1. 5 µL of each culture were seeded on SCD –WL plates and SCD –WL, –Histidine (H) plates + 1 mM 3AT (3-amino-1,2,4-triazole) to select for HIS3 expression. β-galactosidase assay was performed using the yeast β-galactosidase assay kit (Thermo Scientific).

### Protein Interaction Studies

*Drosophila* S2 cells (Invitrogen) cultured at 25°C were transfected with FuGene HD (Roche), incubated for 3 d, and lysed in 1 mL of IP buffer (60 mM Tris HCl, pH 8.0, 1% Tergitol type NP-40 (Sigma), 10% glycerol, 1×Complete protease inhibitor cocktail (Roche), 1×PhoStop phosphatase inhibitor cocktail (Roche)). 100 µL of lysate was stored on ice as input. Immunoprecipitation was performed with the remaining lysate for 2 h at 4°C, using goat anti-myc-conjugated agarose beads (Genetex). Immunoprecipitated complexes were released from the beads with 100 µL of sample buffer (same as IP buffer with the addition of 2% sodium dodecylsulfate (SDS), 0.1 M Dithiothreitol (DTT), and 0.01% bromophenol blue). Samples were analyzed on NuPage 4%–12% Bis-Tris gels (Invitrogen). Immunoblotting was performed using rat monoclonal anti-HA 3F10 coupled to horseradish peroxidase (HRP) (Roche), 1∶2,000; rabbit polyclonal anti-myc (AbCam), 1∶5,000; And goat polyclonal anti-rabbit (Pierce) coupled to HRP, 1∶2,000.

## Supporting Information

Figure S1 Amphid sensory compartment morphogenesis in wild-type embryos. Electron micrographs of cross-sections through the amphid primordium in wild-type animals. Top: At approximately 380 min after fertilization, the amphid pocket is blocked anteriorly by a cap formed by the sheath glia (left). More posteriorly (middle and right), the sheath wraps around the dendrites of the amphid neurons. Bottom: At approximately 400 min after fertilization, the amphid channel is open, with filaments (asterisk) visible at the level of the socket (left; arrow indicates socket self junction). More posteriorly (middle and right), the sheath glia wraps around the dendrites of the amphid neurons. Filaments (asterisk) can be seen in the middle section.
(TIF)

Figure S2 Dye-filling assay and *lit-1*(*ns132*) mapping and cloning. (A–C) Fluorescence images of (A) wt, (B) *daf-6*(*e1377*), and (C) *lit-1*(*ns132*); *daf-6*(*e1377*) animals after incubation for 1.5 h in 10 µg/mL of DiI (red). Scale bars, 50 µm. (D) Using SNP mapping (see Supplemental Materials and Methods, Text S1), *ns132* was mapped to the right end of chromosome III, distal to the SNP F54F12:17329 at genetic position +20.72. The cosmids ZK520, ZK525, W96F12, and K08E3 were used for the construction of transgenic strains (see panel E). (E) Dye-filling in animals of the indicated genotypes (*n*≥90). The alleles used were *daf-6*(*e1377*) and *lit-1*(*ns132*). *lit-1* genomic and *lit-1*(*ns132*) genomic correspond to constructs pGO1 and pGO2, respectively (see Supplemental Materials and Methods, Text S1).
(TIF)

Figure S3 Nuclear localization of LIT-1 is not abrogated by disruption of the LIT-1 carboxy-terminal domain. (A–C) Fluorescence images of sheath glia cell body and nucleus in animals transgenic for the indicated GFP::LIT-1 fusion protein. Transgenes depicted: *nsEx2606* (A), *nsEx2609* (B), *nsEx2747* (C). Arrow, cell nucleus. Scale bar, 10 µm. The *T02B11.3* promoter was used to drive all constructs.
(TIF)

Figure S4 Sensory compartment localization of LIT-1, MOM-4, and ACT-4 are independent of *daf-6*. (A–C) Fluorescence images of adult *daf-6*(*n1543*) animals expressing the indicated GFP fusion proteins. The *T02B11.3* amphid sheath promoter was used to drive all constructs. Trangenes depicted: *nsEx2606* (A), *nsEx2840* (B), *nsEx2876* (C). Anterior is to the left. Scale bars, 10 µm.
(TIF)

Figure S5 Overexpression of LIT-1 within the sheath glia disrupts cellular morphology. (A) Fluorescence projection image of the sheath glia promoter *F16F9.3* driving dsRed (transgene *nsEx3272*). (B) Fluorescence projection image of a transgenic animal carrying a high copy number of the *T02B11.3* amphid sheath promoter driving GFP::LIT-1 (transgene *nsEx2619*). Compare the extensive branching of the sheath glia process with (B). (C) Fluorescence image of the sensory compartment of an animal with the same genotype as the one in (B). Compare with Figure 6A. Anterior is to the left. Scale bars, 10 µm.
(TIF)

Table S1 Components of the Wnt signaling pathway do not affect amphid morphogenesis.
(DOC)

Table S2 Clones identified from a yeast-two-hybrid screen for proteins that interact with the carboxy-terminal domain of LIT-1.
(DOC)

Text S1 Supplemental Materials and Methods.
(DOC)

## Abbreviations

Dyf: dye-filling defective
EM: electron microscopy
EMS: ethyl methanesulfonate
fEM: Fluorescence Electron Microscopy
GFAP: glial fibrillary acidic protein
NLK: Nemo-like kinase
NLS-RFP: nuclearly localized dsRed fluorescent protein
PALM: photo-activated localization microscopy
SDS: sodium-dodecylsulfate
SNP: single nucleotide polymorphism
WASP: Wiskott-Aldrich syndrome protein
WIP: WASP interacting protein
