# Supplementary figures and images for: Opposing Activities of LIT-1/NLK and DAF-6/Patched-Related Direct Sensory Compartment Morphogenesis in C. elegans
Source: PLoS Biol. 2011 Aug 9;9(8):e1001121. doi: 10.1371/journal.pbio.1001121 (PMC3153439; doi:10.1371/journal.pbio.1001121)

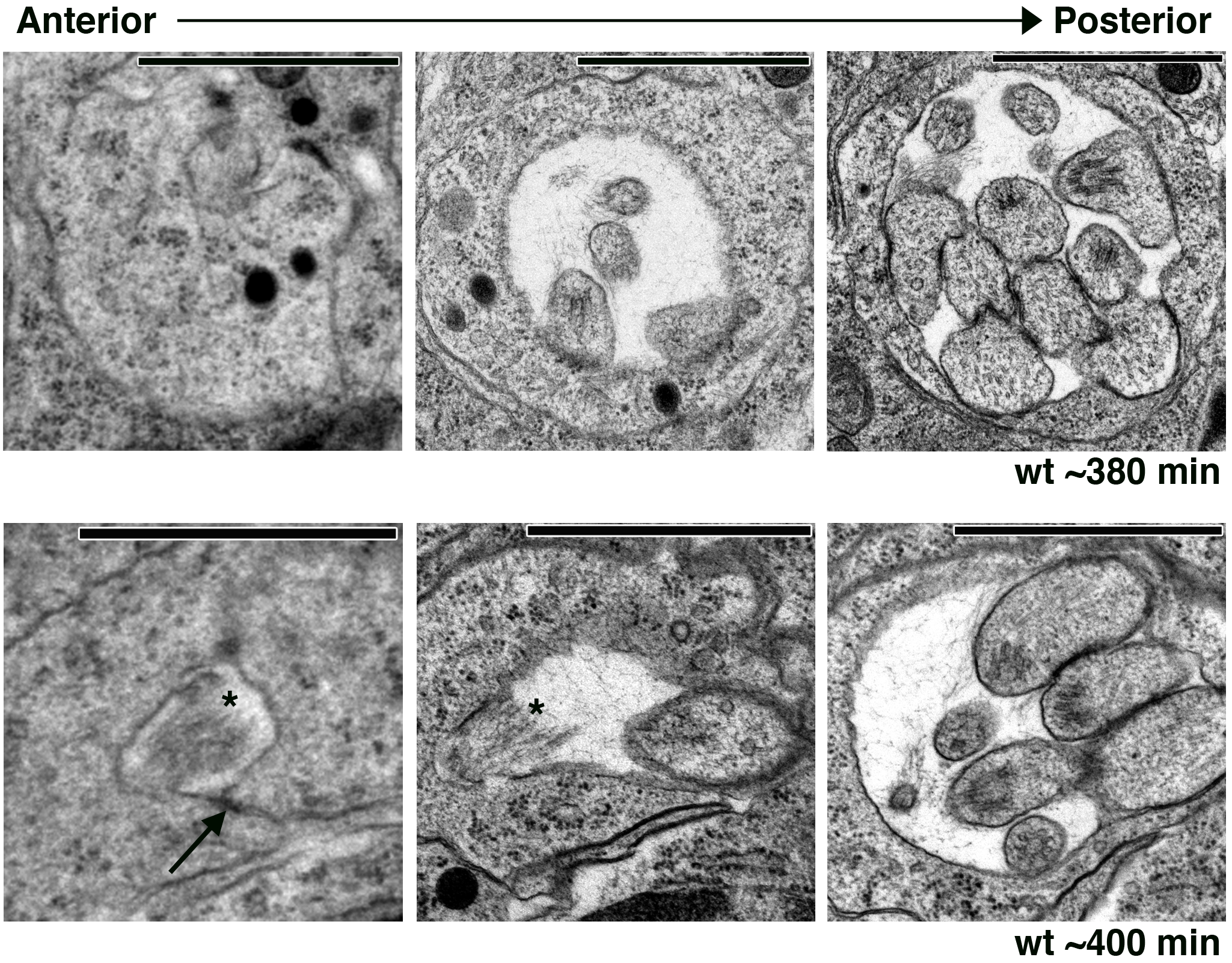

Supplement: Figure S1 — Amphid sensory compartment morphogenesis in wild-type embryos. Electron micrographs of cross-sections through the amphid primordium in wild-type animals. Top: At approximately 380 min after fertilization, the amphid pocket is blocked anteriorly by a cap formed by the sheath glia (left). More posteriorly (middle and right), the sheath wraps around the dendrites of the amphid neurons. Bottom: At approximately 400 min after fertilization, the amphid channel is open, with filaments (asterisk) visible at the level of the socket (left; arrow indicates socket self junction). More posteriorly (middle and right), the sheath glia wraps around the dendrites of the amphid neurons. Filaments (asterisk) can be seen in the middle section. (TIF) [file pbio.1001121.s001.tif]

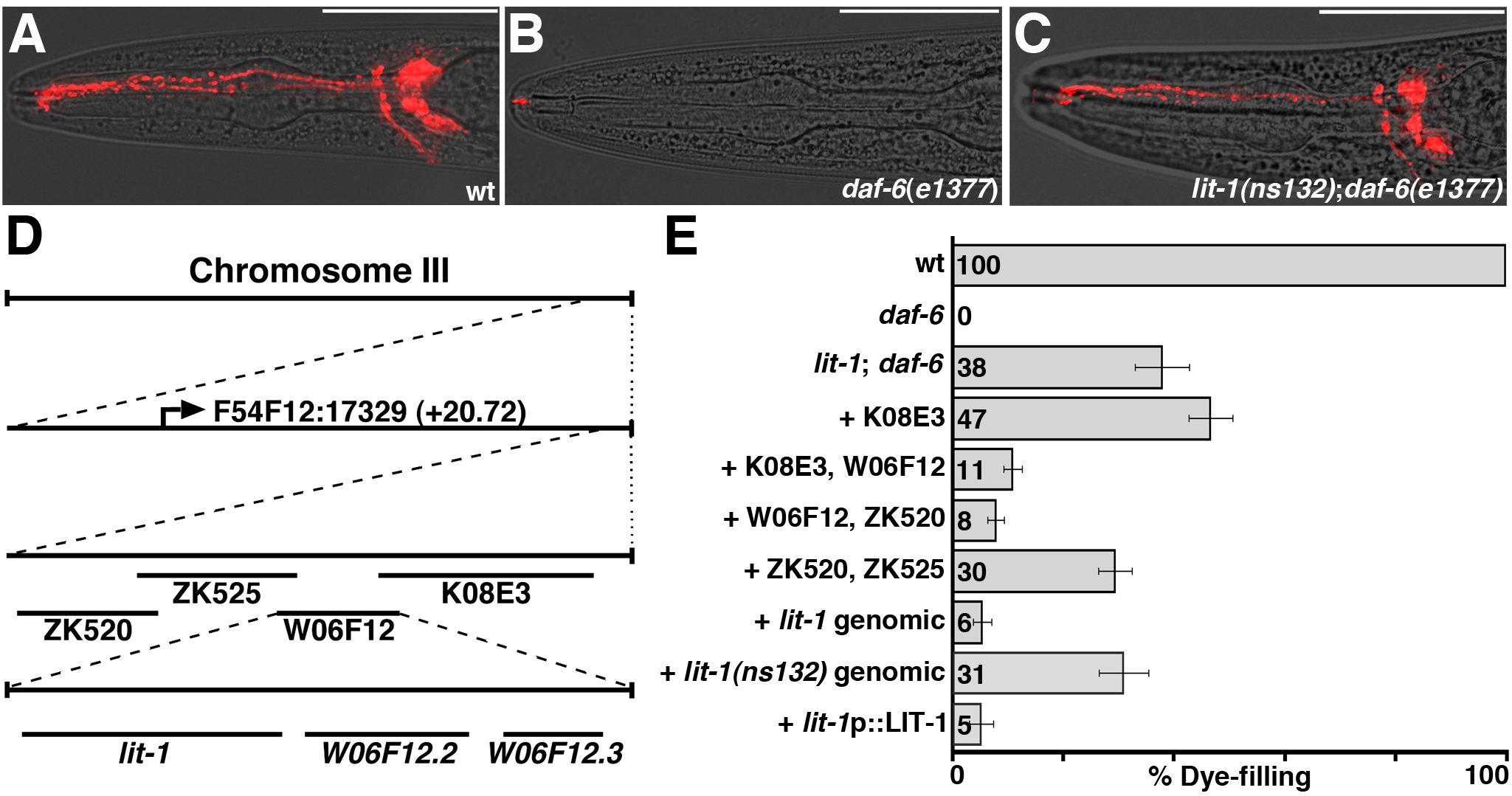

Supplement: Figure S2 — Dye-filling assay and lit-1(ns132) mapping and cloning. (A–C) Fluorescence images of (A) wt, (B) daf-6(e1377), and (C) lit-1(ns132); daf-6(e1377) animals after incubation for 1.5 h in 10 µg/mL of DiI (red). Scale bars, 50 µm. (D) Using SNP mapping (see Supplemental Materials and Methods, Text S1), ns132 was mapped to the right end of chromosome III, distal to the SNP F54F12:17329 at genetic position +20.72. The cosmids ZK520, ZK525, W96F12, and K08E3 were used for the construction of transgenic strains (see panel E). (E) Dye-filling in animals of the indicated genotypes (n≥90). The alleles used were daf-6(e1377) and lit-1(ns132). lit-1 genomic and lit-1(ns132) genomic correspond to constructs pGO1 and pGO2, respectively (see Supplemental Materials and Methods, Text S1). (TIF) [file pbio.1001121.s002.tif]

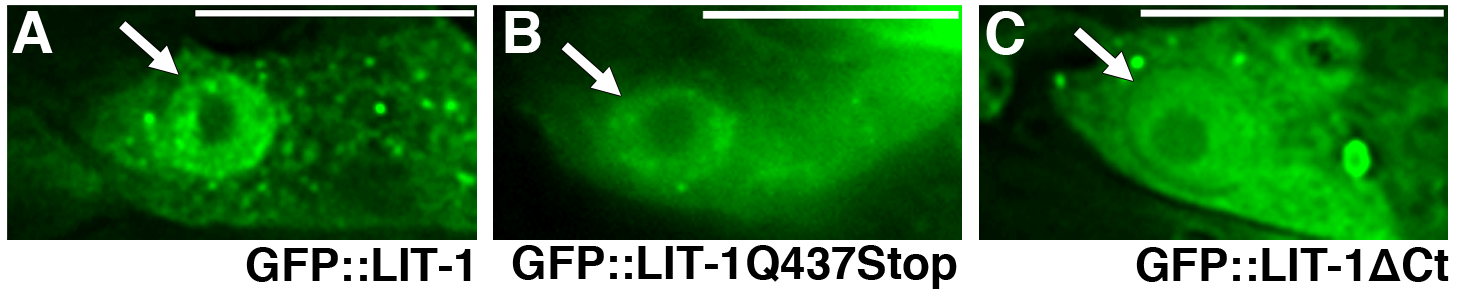

Supplement: Figure S3 — Nuclear localization of LIT-1 is not abrogated by disruption of the LIT-1 carboxy-terminal domain. (A–C) Fluorescence images of sheath glia cell body and nucleus in animals transgenic for the indicated GFP::LIT-1 fusion protein. Transgenes depicted: nsEx2606 (A), nsEx2609 (B), nsEx2747 (C). Arrow, cell nucleus. Scale bar, 10 µm. The T02B11.3 promoter was used to drive all constructs. (TIF) [file pbio.1001121.s003.tif]

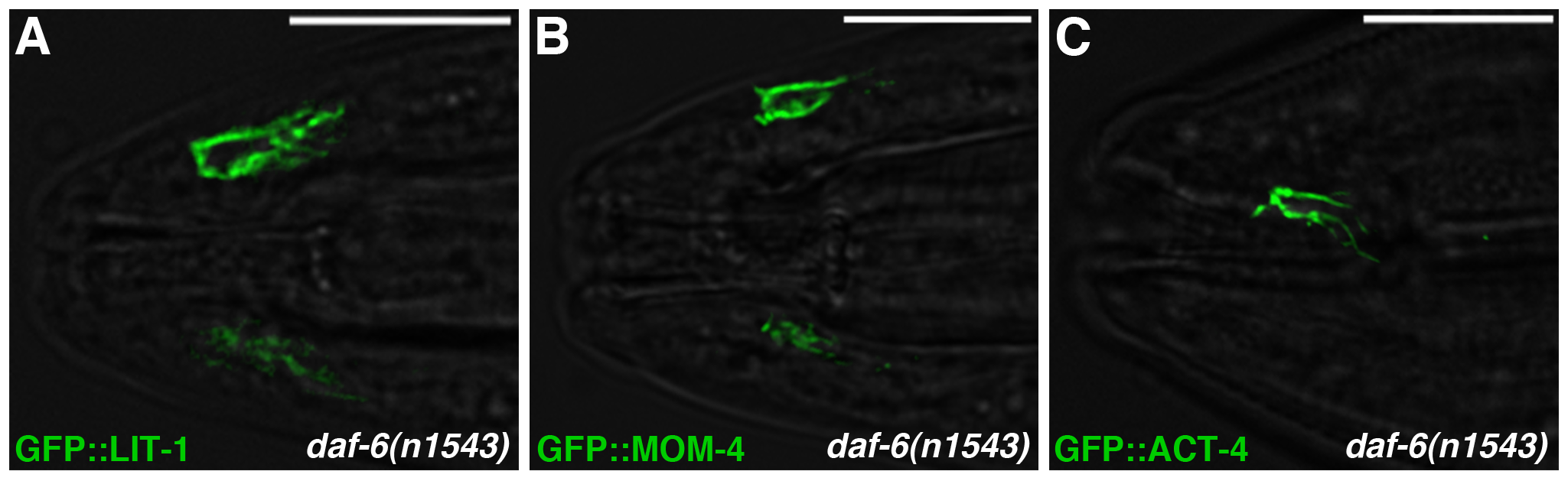

Supplement: Figure S4 — Sensory compartment localization of LIT-1, MOM-4, and ACT-4 are independent of daf-6. (A–C) Fluorescence images of adult daf-6(n1543) animals expressing the indicated GFP fusion proteins. The T02B11.3 amphid sheath promoter was used to drive all constructs. Trangenes depicted: nsEx2606 (A), nsEx2840 (B), nsEx2876 (C). Anterior is to the left. Scale bars, 10 µm. (TIF) [file pbio.1001121.s004.tif]

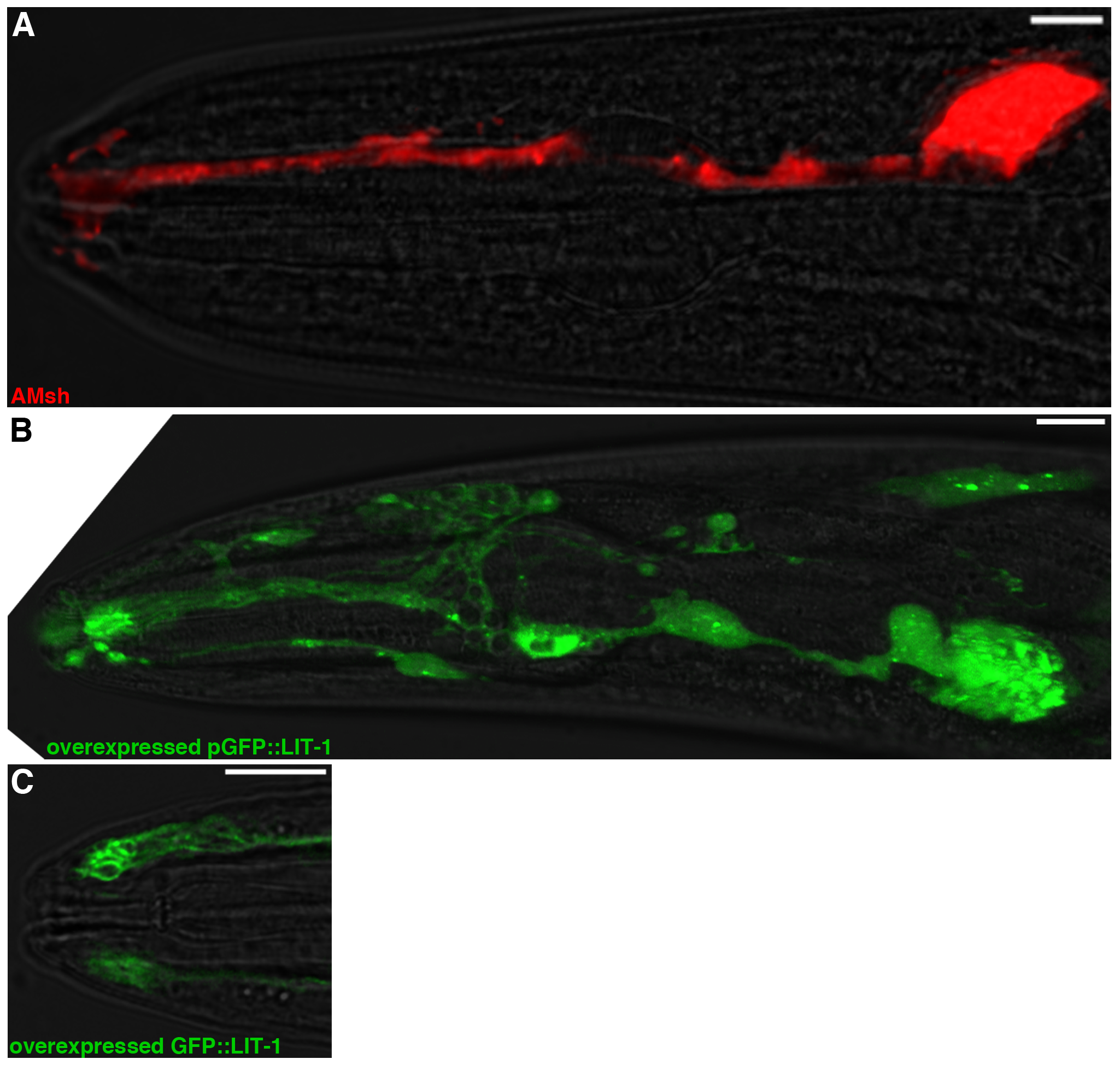

Supplement: Figure S5 — Overexpression of LIT-1 within the sheath glia disrupts cellular morphology. (A) Fluorescence projection image of the sheath glia promoter F16F9.3 driving dsRed (transgene nsEx3272). (B) Fluorescence projection image of a transgenic animal carrying a high copy number of the T02B11.3 amphid sheath promoter driving GFP::LIT-1 (transgene nsEx2619). Compare the extensive branching of the sheath glia process with (B). (C) Fluorescence image of the sensory compartment of an animal with the same genotype as the one in (B). Compare with Figure 6A. Anterior is to the left. Scale bars, 10 µm. (TIF) [file pbio.1001121.s005.tif]
