# Supplementary material for: Opposing Activities of LIT-1/NLK and DAF-6/Patched-Related Direct Sensory Compartment Morphogenesis in C. elegans
Source: PLoS Biol. 2011 Aug 9;9(8):e1001121. doi: 10.1371/journal.pbio.1001121 (PMC3153439; doi:10.1371/journal.pbio.1001121)
Supplement: Table S1 — Components of the Wnt signaling pathway do not affect amphid morphogenesis. (DOC) [file pbio.1001121.s006.doc]

| **Table S1. Components of the Wnt signaling pathway do not affect amphid morphogenesis** | | | | |
| --- | --- | --- | --- | --- |
| Common name | *C .elegans* gene name | Allele | % Dye-filling^a^ | % Dye-filling in  *daf-6*(*n1543*)^b^ |
| Porcupine | *mom-1* | RNAi | ND^c^ | 0 |
| Wntless | *mig-14* | *mu71* | 100 | 5 |
|  |  | *ga62* | 100 | 2 |
| Wnt | *lin-44* | *n1792* | 100 | 0 |
|  | *egl-20* | *mu27* ^d^ | 100 | 0 |
|  | *cwn-1* | *ok546* | 100 | 3 |
|  | *cwn-2* | *ok895* | 100 | 0 |
|  | *mom-2* | *ne834* | 100 | 0 |
|  |  | *or309* | 100 | ND |
| Frizzled | *lin-17* | *n671* | 100 | 4 |
|  |  | *n698* | 100 | ND |
|  |  | *n3091* | 100 | ND |
|  | *mig-1* | *e1787* | 100 | 6 |
|  | *mom-5* | *or57, zu193* | 100 | ND |
|  |  | RNAi | ND | 0 |
|  | *cfz-2* | *ok1201* | 100 | 5 |
|  | *mig-1 lin-17; cfz-2* | *e1787, n677, ok1201* respectively | 100 | 0 |
| Dishevelled | *mig-5* | *rh147* | 100 | 0 |
|  | *dsh-1* | *ok1445* | 100 | 0 |
|  |  | RNAi | ND | 0 |
|  | *dsh-2* | *ok2162* | 100 | ND |
|  |  | RNAi | ND | 0 |
| Ryk | *lin-18* | *e620* | 100 | 0 |
| β-catenin | *bar-1* | *ga80* | 100 | 2 |
|  | *wrm-1* | *ne1982* | 100 | 1 |
|  | *hmp-2* | RNAi | ND | 0 |
|  | *sys-1* | *q544* | 100 | ND |
|  |  | RNAi | ND | 0 |
| TCF/LEF | *pop-1* | *q624* | 100 | 0 |
|  |  | RNAi | ND | 0 |
| ROR1 | *cam-1* | *ks52* | 100 | 8 |
| Van Gogh/  Strabismus | *vang-1* | *ok1142* | 100 | 0 |

^a^ n≥100 for each genotype.

^b^ Full genetic background was *unc-3*(*e151*) *daf-6*(*n1543*) except for RNAi experiments where background was *rrf-3*(*pk1426*); *unc-3*(*e151*)*daf-6*(*n1543*). *rrf-3* increases the sensitivity to RNAi [1], but does not affect dye-filling (data not shown); n≥100 for all experiments.

^c^ ND, not determined.

^d^ The reference *egl-20* allele *n585* harbors a background mutation that suppresses the dye-filling defects of *daf-6*(*n1543*). The *mu27* allele, shown here, has the same molecular lesion as *n585* [2], but does not suppress *daf-6*.

1. Simmer F, Tijsterman M, Parrish S, Koushika SP, Nonet ML et al. (2002) Loss of the putative RNA-directed RNA polymerase RRF-3 makes C. elegans hypersensitive to RNAi. Curr Biol 12: 1317-1319.

2. Maloof JN, Whangbo J, Harris JM, Jongeward GD, Kenyon C (1999) A Wnt signaling pathway controls hox gene expression and neuroblast migration in C. elegans. Development 126: 37-49.
