# Supplementary material for: Opposing Activities of LIT-1/NLK and DAF-6/Patched-Related Direct Sensory Compartment Morphogenesis in C. elegans
Source: PLoS Biol. 2011 Aug 9;9(8):e1001121. doi: 10.1371/journal.pbio.1001121 (PMC3153439; doi:10.1371/journal.pbio.1001121)
Supplement: Table S2 — Clones identified from a yeast-two-hybrid screen for proteins that interact with the carboxy-terminal domain of LIT-1. (DOC) [file pbio.1001121.s007.doc]

| **Table S2. Clones identified from a yeast-two-hybrid screen for proteins that interact with the carboxy-terminal domain of LIT-1** | | |
| --- | --- | --- |
| **Gene** | **No. clones found** | **Description** |
| *mep-1* | 1 | zinc-finger protein |
| T24E12.9 | 1 | protein of unknown function |
| *vit-3* | 2 | vitellogenin |
| *fbp-1* | 1 | fructose 1,6-bisphosphatase |
| *act-4* | 4 | actin |
| Y87G2A.1 | 2 | protein of unknown function |
| *wsp-1* | 1 | WASP |
| *ztf-16* | 1 | zinc-finger protein |
| C50F4.1 | 1 | protein of unknown function |
| C44B12.5 | 6 | protein of unknown function |
| *nrde-3* | 1 | argonaute protein |
| *ost-1* | 1 | osteonectin, ECM protein |
| *unc-52* | 1 | perlecan, ECM protein |
| *tag-30* | 1 | protein of unknown function |
| *vit-4* | 1 | vitellogenin |
| C34F11.3 | 1 | adenosine monophosphate deaminase |
