# Supplementary material for: Opposing Activities of LIT-1/NLK and DAF-6/Patched-Related Direct Sensory Compartment Morphogenesis in C. elegans
Source: PLoS Biol. 2011 Aug 9;9(8):e1001121. doi: 10.1371/journal.pbio.1001121 (PMC3153439; doi:10.1371/journal.pbio.1001121)
Supplement: Text S1 — Supplemental Materials and Methods. (DOC) [file pbio.1001121.s008.doc]

**Supplemental Materials and Methods**

**Strains**

Strains were handled using standard methods [1]. All strains were maintained and scored at 20°C unless otherwise indicated. The alleles used in this study are: *daf-6*(*e1377*, *n1543*) [2] and [3] respectively), *lit-1*(*t1512*) [4], *lit-1*(*ns132*) (described here), *che-14*(*ok193*) [5], *wsp-1*(*gm324*) [6], *mom-4*(*ne1539*)[7], a gift from Craig Mello), *daf-19*(*m86*) [8], *daf-16*(*mu86*)( [9], *mig-14*(mu71) [10], *mig-14*(*ga62*)[11], *lin-44*(*n1792*) [12], *egl-20*(*n585*, *mu27*) [13] and [10] respectively), *cwn-1*(*ok546*) [14], *cwn-2*(*ok895*) [14], *mom-2*(*ne834*) [7], a gift from Craig Mello), *mom-2*(*or309*) [15], *lin-17*(*n671*, *n698*) [16], *lin-17*(*n3091*) [17], *mig-1*(*e1787*) [18], *mom-5*(*or57*) [15], *mom-5*(*zu193*) [19], *cfz-2*(*ok1201*) [14], *mig-5*(*rh147*) [20], *lin-18*(*e620*) [16], *bar-1*(*ga80*)) [11], *wrm-1*(*ne1982*) [7], a gift from Craig Mello), *pop-1*(*q624*) [21], *cam-1*(*ks52*) [22], *vang-1*(*ok1142*) [23], *unc-*3(*e151*) [24], *unc-32*(*e189*) [25].

Unstable extrachromosomal transgenes used in this study:

| **Extrachromosomal array(s)** | **Constructs** |
| --- | --- |
| *nsEx1933*, *nsEx1934*, *nsEx1935, nsEx1936* | pGO1, pMH135 |
| *nsEx1931*, *nsEx1932* | pGO2, pMH135 |
| *nsEx2159*, *nsEx2160* | pGO6, *ptr-10*pro::NLSRFP, pRF4 |
| *nsEx2078*, *nsEx2079*, *nsEx2080*, *nsEx2081* | pGO8, pMH135 |
| *nsEx2108*, *nsEx2109*, *nsEx2110*, *nsEx2111*, *nsEx2112*, *nsEx2113* | pGO10, *vap-1*pro::GFP |
| *nsEx2308*, *nsEx2309*, *nsEx2310* | pGO17, *vap-1*pro::GFP, pRF4 |
| *nsEx2952*, *nsEx2953*, *nsEx2954* | pGO18, pMH135 |
| *nsEx2541*, *nsEx2542*, *nsEx2543* | pGO20, pEP51 |
| *nsEx2539*, *nsEx2540* | pGO32, pEP51 |
| *nsEx2605*, *nsEx2606*, *nsEx2607*, *nsEx2608, nsEx2619*, *nsEx2829*, *nsEx2830*, *nsEx2831* | pGO38, pRF4 |
| *nsEx2609*, *nsEx2610*, *nsEx2611*, *nsEx2612* | pGO47, pRF4 |
| *nsEx2626*, *nsEx2627*, *nsEx2628*, *nsEx2629*, | pGO56, pRF4 |
| *nsEx2747*, *nsEx2748*, *nsEx2749* | pGO73, pRF4 |
| *nsEx2760*, *nsEx2761*, *nsEx2762*, *nsEx2766*, *nsEx2767*, *nsEx2768*,  *nsEx2750*, *nsEx2751*,*nsEx2752* | *T02B11.3*pro::GFP, *gcy-5*pro::mCherry, pEP51 |
| *nsEx2838*, *nsEx2839*, *nsEx2840* | pGO91, pRF4 |
| *nsEx2874*, *nsEx2875*, *nsEx2876* | pGO116, pGO93 |
| *nsEx2968*, *nsEx2969*, *nsEx2970* | pGO120, pRF4 |
| *nsEx3243*, *nsEx3244*, *nsEx3245*, *nsEx3246* | pGO177, pGO65, pRF4 |

**Plasmid Construction**

Table of the plasmids used in this study. All pGO constructs were made using pPD95.75 (Andrew Fire) as a backbone, unless otherwise noted.

| **Plasmid** | **Description** | **Details** |
| --- | --- | --- |
| pGO1 | *lit-1* genomic region | 8.2 kb genomic region that includes the lit-1 locus (W06F12.1b.1 transcript) with a 2.1 kb promoter region and a 637 bp 3’UTR (SalI/AflII)  Forward primer: gtcgaccgattttttttcacg  Reverse primer: gtgaaagaactcggtagtattggcac |
| pGO2 | *lit-1*(*ns132*) genomic region | Same as pGO1 but amplified from the *lit-1*(*ns132*) strain |
| pGO6 | *lit-1*pro::NLS-GFP | *lit-1*pro consists of 2.5 kb upstream of the *lit-1* start site (W06F12.1b.1 transcript) (SphI/BamHI). Cloned in pPD95.69 (Andrew Fire) |
| pGO8 | *lit-1*pro::LIT-1 | *lit-1* cDNA (yk1457b04) a gift from Yuji Kohara (AgeI/EcoRI) |
| pGO10 | *lin-26myo-2*pro::LIT-1 | The e1 *lin-26* promoter fragment [26] fused to the *myo-2* minpro [27] (a gift from Maxwell G. Heiman [28] (SphI/XbaI), |
| pGO17 | *lit-1*pro::NLS-RFP | see pGO6 |
| pGO18 | *dyf-7*pro::LIT-1 | *dyf-7*pro (SphI/XmaI) a gift from Maxwell G. Heiman [28], driving the *lit-1* cDNA (see pGO10) |
| pGO20 | *lin-26myo-2*pro::GFP::LIT-1 | *lin-26myo-2*pro (see pGO10) driving a rescuing GFP::LIT-1 fusion |
| pGO32 | *vap-1*pro::GFP::LIT-1 | *vap-1*pro a gift from Leo Liu. See also [29] |
| pGO38 | *T02B11.3*pro::GFP::LIT-1 | *T02B11.3*pro a gift from Maya Tevlin [30] |
| pGO47 | *T02B11.3*pro::GFP::LIT-1Q437Stop | The *lit-1* cDNA truncated at Q437 |
| pGO56 | *T02B11.3*pro::GFP::LIT-1Ct | GFP fused to the carboxy-terminal domain of LIT-1 (last 103aa, EEGRLRFH...PPSPQAW) |
| pGO65 | *F16F9.3*pro::mCherry::LIT-1 | *F16F9.3*pro a gift from Maya Tevlin [31] |
| pGO73 | *T02B11.3*pro::GFP::LIT-1ΔCt | GFP fused to *lit-1* cDNA truncated at L359 |
| pGO87 | pLexA-N::LIT-1Ct | LexA fused to the carboxy-terminal domain of LIT-1 (last 103aa, EEGRLRFH...PPSPQAW) |
| pGO91 | *T02B11.3*pro::GFP::MOM-4 | GFP fused to *mom-4* cDNA (yk1072f05), a gift from Yuji Kohara |
| pGO93 | *pha-4*pro::mCherry | *pha-4*pro a gift from Maxwell G. Heiman |
| pGO116 | *T02B11.3*pro::GFP::ACT-4 | GFP fused to *act-4* cDNA |
| pGO119 | *Ac*::MYC::WSP-1 | myc tagged *wsp-1* cDNA in the pAc, Drosophila actin 5c promoter vector, a gift from Michael Chiorazzi; see [32] |
| pGO120 | T02B11.3pro::mEos::ACT-4 | mEos a gift from Loren L Looger. See [33] |
| pGO123 | *Ac::*HA::eGFP::LIT-1 | See pGO119. eGFP a gift from Maya Bader |
| pGO131 | *T02B11.3*pro::GFP::ACT-1 | *T02B11.3*pro a gift from Maya Tevlin [30] |
| pGO177 | *T02B11.3*pro::GFP::WSP-1 | *T02B11.3*pro a gift from Maya Tevlin [30] |
| pRF4 | *rol-6*(*su1006*) | from [34] |
| pMH135 | *pha-4*pro::GFP | a gift from Maxwell G. Heiman [28] |
| pEP51 | *unc-122*pro::GFP | coelomocyte marker |
|  | *ptr-10*pro::NLS-RFP | from [35] |
|  | *vap-1*pro::GFP | *vap-1*pro a gift from Leo Liu. See also [29] |
|  | *gcy-5*pro::mCherry | *gcy-5*pro after [36] |
|  | *T02B11.3*pro::GFP | a gift from Maya Tevlin [30] |
|  | *F16F9.3*pro::mCherry | a gift from Maya Tevlin [31] |

***lit-1* Mapping and Cloning**

*ns132* was mapped using single nucleotide polymorphism mapping [37] to the right arm of Chromosome III. We generated transgenic *ns132*; *daf-6(e1377)* animals carrying extrachromosomal arrays of cosmids from this region (provided by the Sanger Center, Cambridge, UK). The genes in the rescuing cosmid, W06F12, were sequenced, and a C->T transition creating a premature stop codon was identified in the last exon of the *lit-1* gene.

**Transmission Electron Microscopy (EM)**

Previously described conventional fixation methods were used for adult animals [29]. High-pressure fixation was used for embryos and some adult animals. Briefly, samples were frozen using the Leica High Pressure Freezer EM-PACT2 (pressure of 18,000 bar, cooling rate of 20,000 °C/sec). Freeze substitution was performed using the Leica EM AFS2 Automatic Freeze Substitution System [38]. Ultrathin serial sections (60 nm) were cut using a REICHERT Ultra-Cut-E ultramicrotome and collected on Pioloform-coated single-slot copper grids. EM images for every other section were acquired using an FEI Tecnai G2 Spirit BioTwin transmission electron microscope operating at 80 kV with a Gatan 4K x 4K digital camera.

**Fluorescence Electron Microscopy (fEM)**

Sample preparation: *C. elegans* animals expressing mEos2::ACT-4 [33] were prepared for fEM as previously described [39]. Transgenic animals were raised in the dark and adults were rapidly frozen together with bacteria, as a cryoprotectant, using a high-pressure freezer (Bal-Tec, HM010). Frozen samples were transferred under liquid nitrogen into cryovials containing 0.1% potassium permanganate (EMS) + 0.001% osmium tetroxide (EMS, crystals) in 95% acetone. Freeze-substitution and subsequent plastic embedding were carried out in an automated freeze-substitution unit as follows: -90°C for 30 h, 5°C/h to -30°C, -30°C for 2 h for the freeze-substitution and -30°C for 48 h for the plastic embedding. Fixatives were washed out with 95% ethanol 6 times over 2 h. Animals were then infiltrated with glycol methacrylate (GMA) solutions in three steps: 30% for 5 h, 70% for 6 h, and 100% overnight. Specimens were moved to a cap of polypropylene BEEM capsules (EBSciences), and the plastic media was exchanged with freshly mixed and pre-cooled GMA three times over a period of 6 h. At the last step of the exchange, animals were separated from the bacteria using tweezers (EMS, #5), and GMA media containing 0.15% of N,N-Dimethyl-p-toluidine (Sigma-Aldrich) was added for polymerization. Polymerization was complete after 12 h. Plastic blocks were stored in a vacuum bag at -20°C until imaging.

Protein localization by fEM [39]: Serial sections (80 nm) were collected onto pre-cleaned coverslips. For fluorescence nanoscopy, photo-activated localization microscopy (PALM; Zeiss, PAL-M, Prototype Serial No. 2701000005) was employed. The region of interest was screened using wide-field illumination. Just prior to PALM imaging, 250 nm gold nanoparicles (Micospheres-Nanospheres), which serve as fiduciary markers, were applied to the sections for 4 min. Then, 3500-5000 frames with an exposure time of 50 ms/frame were collected while stochastically photo-converting mEos signals with 1 µW of a 405 nm laser. For EM imaging, sections were then stained with 2.5% uranyl acetate (EMS) in water, and a thin layer of carbon was applied. Back-scattered electrons were collected using a scanning electron microscope (FEI, nova nano) and a high contrast solid-state detector (FEI, vCD). Fluorescence and electron micrographs were aligned based on the gold fiduciary markers.

1. Brenner S (1974) The genetics of Caenorhabditis elegans. Genetics 77: 71-94.

2. Riddle DL, Swanson MM, Albert PS (1981) Interacting genes in nematode dauer larva formation. Nature 290: 668-671.

3. Starich TA, Herman RK, Kari CK, Yeh WH, Schackwitz WS et al. (1995) Mutations affecting the chemosensory neurons of Caenorhabditis elegans. Genetics 139: 171-188.

4. Kaletta T, Schnabel H, Schnabel R (1997) Binary specification of the embryonic lineage in Caenorhabditis elegans. Nature 390: 294-298.

5. Michaux G, Gansmuller A, Hindelang C, Labouesse M (2000) CHE-14, a protein with a sterol-sensing domain, is required for apical sorting in C. elegans ectodermal epithelial cells. Curr Biol 10: 1098-1107.

6. Withee J, Galligan B, Hawkins N, Garriga G (2004) Caenorhabditis elegans WASP and Ena/VASP Proteins Play Compensatory Roles in Morphogenesis and Neuronal Cell Migration. Genetics 167: 1165.

7. Nakamura K, Kim S, Ishidate T, Bei Y, Pang K et al. (2005) Wnt signaling drives WRM-1/beta-catenin asymmetries in early C. elegans embryos. Genes Dev 19: 1749-1754.

8. Perkins LA, Hedgecock EM, Thomson JN, Culotti JG (1986) Mutant sensory cilia in the nematode Caenorhabditis elegans. Dev Biol 117: 456-487.

9. Lin K, Hsin H, Libina N, Kenyon C (2001) Regulation of the Caenorhabditis elegans longevity protein DAF-16 by insulin/IGF-1 and germline signaling. Nat Genet 28: 139-145.

10. Harris J, Honigberg L, Robinson N, Kenyon C (1996) Neuronal cell migration in C. elegans: regulation of Hox gene expression and cell position. Development 122: 3117-3131.

11. Eisenmann DM, Kim SK (2000) Protruding vulva mutants identify novel loci and Wnt signaling factors that function during Caenorhabditis elegans vulva development. Genetics 156: 1097-1116.

12. Herman MA, Horvitz HR (1994) The Caenorhabditis elegans gene lin-44 controls the polarity of asymmetric cell divisions. Development 120: 1035-1047.

13. Trent C, Tsuing N, Horvitz HR (1983) Egg-laying defective mutants of the nematode Caenorhabditis elegans. Genetics 104: 619-647.

14. Zinovyeva AY, Forrester WC (2005) The C. elegans Frizzled CFZ-2 is required for cell migration and interacts with multiple Wnt signaling pathways. Dev Biol 285: 447-461.

15. Thorpe CJ, Schlesinger A, Carter JC, Bowerman B (1997) Wnt signaling polarizes an early C. elegans blastomere to distinguish endoderm from mesoderm. Cell 90: 695-705.

16. Ferguson EL, Horvitz HR (1985) Identification and characterization of 22 genes that affect the vulval cell lineages of the nematode Caenorhabditis elegans. Genetics 110: 17-72.

17. Sawa H, Lobel L, Horvitz HR (1996) The Caenorhabditis elegans gene lin-17, which is required for certain asymmetric cell divisions, encodes a putative seven-transmembrane protein similar to the Drosophila Frizzled protein. Genes Dev 10: 2189-2197.

18. Desai C, Garriga G, McIntire SL, Horvitz HR (1988) A genetic pathway for the development of the Caenorhabditis elegans HSN motor neurons. Nature 336: 638-646.

19. Rocheleau CE, Downs WD, Lin R, Wittmann C, Bei Y et al. (1997) Wnt signaling and an APC-related gene specify endoderm in early C. elegans embryos. Cell 90: 707-716.

20. Walston T, Guo C, Proenca R, Wu M, Herman M et al. (2006) mig-5/Dsh controls cell fate determination and cell migration in C. elegans. Dev Biol 298: 485-497.

21. Siegfried KR, Kimble J (2002) POP-1 controls axis formation during early gonadogenesis in C. elegans. Development 129: 443-453.

22. Koga M, Take-uchi M, Tameishi T, Ohshima Y (1999) Control of DAF-7 TGF-β expression and neuronal process development by a receptor tyrosine kinase KIN-8 in Caenorhabditis elegans. Development 126: 5387-5398.

23. Green JL, Inoue T, Sternberg PW (2008) Opposing Wnt pathways orient cell polarity during organogenesis. Cell 134: 646-656.

24. Prasad BC, Ye B, Zackhary R, Schrader K, Seydoux G et al. (1998) unc-3, a gene required for axonal guidance in Caenorhabditis elegans, encodes a member of the O/E family of transcription factors. Development 125: 1561-1568.

25. Pujol N, Bonnerot C, Ewbank JJ, Kohara Y, Thierry-Mieg D (2001) The Caenorhabditis elegans unc-32 gene encodes alternative forms of a vacuolar ATPase a subunit. J Biol Chem 276: 11913-11921.

26. Landmann F, Quintin S, Labouesse M (2004) Multiple regulatory elements with spatially and temporally distinct activities control the expression of the epithelial differentiation gene lin-26 in C. elegans. Dev Biol 265: 478-490.

27. Okkema PG, Harrison SW, Plunger V, Aryana A, Fire A (1993) Sequence requirements for myosin gene expression and regulation in Caenorhabditis elegans. Genetics 135: 385-404.

28. Heiman MG, Shaham S (2009) DEX-1 and DYF-7 establish sensory dendrite length by anchoring dendritic tips during cell migration. Cell 137: 344-355.

29. Perens EA, Shaham S (2005) C. elegans daf-6 encodes a patched-related protein required for lumen formation. Dev Cell 8: 893-906.

30. Wang Y, Apicella A, Lee SK, Ezcurra M, Slone RD et al. (2008) A glial DEG/ENaC channel functions with neuronal channel DEG-1 to mediate specific sensory functions in C. elegans. EMBO J 27: 2388-2399.

31. Bacaj T, Tevlin M, Lu Y, Shaham S (2008) Glia are essential for sensory organ function in C. elegans. Science 322: 744-747.

32. Han K, Levine MS, Manley JL (1989) Synergistic activation and repression of transcription by Drosophila homeobox proteins. Cell 56: 573-583.

33. Mckinney SA, Murphy CS, Hazelwood KL, Davidson MW, Looger LL (2009) A bright and photostable photoconvertible fluorescent protein. Nat Methods 6: 131.

34. Mello CC, Kramer JM, Stinchcomb D, Ambros V (1991) Efficient gene transfer in C. elegans: extrachromosomal maintenance and integration of transforming sequences. EMBO J 10: 3959-3970.

35. Yoshimura S, Murray JI, Lu Y, Waterston RH, Shaham S (2008) mls-2 and vab-3 control glia development, hlh-17/Olig expression and glia-dependent neurite extension in C. elegans. Development 135: 2263-2275.

36. Yu S, Avery L, Baude E, Garbers DL (1997) Guanylyl cyclase expression in specific sensory neurons: a new family of chemosensory receptors. Proc Natl Acad Sci USA 94: 3384-3387.

37. Wicks SR, Yeh RT, Gish WR, Waterston RH, Plasterk RH (2001) Rapid gene mapping in Caenorhabditis elegans using a high density polymorphism map. Nat Genet 28: 160-164.

38. McDonald K (2007) Cryopreparation methods for electron microscopy of selected model systems. Methods Cell Biol 79: 23-56.

39. Watanabe S, Punge A, Hollopeter G, Willig KI, Hobson RJ et al. (2011) Protein localization in electron micrographs using fluorescence nanoscopy. Nat Methods 8: 80-84.
